# Totality of the Evidence Suggests Prenatal Cannabis Exposure Does Not Lead to Cognitive Impairments: A Systematic and Critical Review

**DOI:** 10.3389/fpsyg.2020.00816

**Published:** 2020-05-08

**Authors:** Ciara A. Torres, Christopher Medina-Kirchner, Kate Y. O'Malley, Carl L. Hart

**Affiliations:** ^1^School of Social Work, Columbia University, New York, NY, United States; ^2^Department of Psychology, Columbia University, New York, NY, United States; ^3^Division on Substance Use, Department of Psychiatry, New York State Psychiatric Institute, New York, NY, United States; ^4^Department of Psychological Sciences, Swinburne University, Hawthorn, VIC, Australia

**Keywords:** marijuana, prenatal, cognition, impairment, normative data

## Abstract

**Background:** Despite limited data demonstrating pronounced negative effects of prenatal cannabis exposure, popular opinion and public policies still reflect the belief that cannabis is fetotoxic.

**Methods:** This article provides a critical review of results from longitudinal studies examining the impact of prenatal cannabis exposure on multiple domains of cognitive functioning in individuals aged 0 to 22 years. A literature search was conducted through PsycINFO, PubMed, and Google Scholar. Articles were included if they examined the cognitive performance of offspring exposed to cannabis *in utero*.

**Results:** An examination of the total number of statistical comparisons (*n* = 1,001) between groups of participants that were exposed to cannabis prenatally and non-exposed controls revealed that those exposed performed differently on a minority of cognitive outcomes (worse on <3.5 percent and better in <1 percent). The clinical significance of these findings appears to be limited because cognitive performance scores of cannabis-exposed groups overwhelmingly fell within the normal range when compared against normative data adjusted for age and education.

**Conclusions:** The current evidence does not suggest that prenatal cannabis exposure alone is associated with clinically significant cognitive functioning impairments.

## Introduction

In the United States (U.S.), and in most countries around the world, cannabis is illegal. Still, according to recent data from the U.S., more than 25 million people reported past month cannabis use, easily outpacing the number of current cocaine (2.2 million) and heroin users (494,000) (NSDUH Detailed Tables, 2017). In addition, 63.8% of respondents to a global drug survey endorsed using cannabis at least once, a rate higher than any other illicit drug (GDS, 2019). Taken together, these findings demonstrate that cannabis use persists in the U.S. as well as around the globe despite legal restrictions.

Recently, countries such as Uruguay and Canada have legalized cannabis for recreational purposes. In the U.S., 11 states have legalized adult cannabis use, while 33 states now allow medical use of the drug. As a result of these recent developments, increased concerns have been raised about cannabis use by pregnant individuals and the impact it may have on the developing fetus. Indeed, cannabis is the most frequently used illicit substance by reproductive aged women in the U.S. (van Gelder et al., 2010; NSDUH Detailed Tables, 2017; National Pregnancy Health Survey, 2019). However, reported use during pregnancy is uncommon (Ko et al., 2015; NSDUH Detailed Tables, 2017; National Pregnancy Health Survey, 2019). Even when cannabis is used by pregnant individuals, use of the drug substantially decreases as pregnancy progresses (Ko et al., 2015). Nonetheless, there remains a minority of women who consume cannabis throughout pregnancy (Ko et al., 2015).

There is a growing scientific database assessing the effects of prenatal cannabis exposure on a myriad of measures, including early physical growth. In general, when proper controls are included, no relationship between prenatal cannabis exposure and adverse physical neonatal outcomes such as birth weight and head circumference has been found (Conner et al., 2016; Grant et al., 2018). Still, a concern expressed in the scientific literature is that although cannabis may not lead to severe physical abnormalities in infants, it might cause subtle changes in the brain that later manifest as deficits in cognitive functioning.

A burgeoning number of reviews have assessed the impact of prenatal cannabis exposure on cognitive functioning (Karila et al., 2006; Wu et al., 2011; Calvigioni et al., 2014; Higuera-Matas et al., 2015). The studies reviewed show that subtle differences in the cognitive performance between children who had been exposed to the drug prenatally and controls do exist, but the conclusions drawn sometimes extend too far beyond the actual data. For example, based on these subtle differences, some researchers have suggested that children prenatally exposed to cannabis exhibit cognitive deficits and/or behavioral abnormalities (Karila et al., 2006; Wu et al., 2011; Calvigioni et al., 2014; Higuera-Matas et al., 2015). The clinical implications of these subtle differences, however, are nearly impossible to determine without knowledge of the expected range of performance for a particular group. Through the use of normative data, whereby individual or mean group scores are compared against a normative database that accounts for age, and educational level, the clinical significance of the differences can be determined. This is a core assessment principle in clinical neuropsychology but appears to be largely ignored in the literature on prenatal cannabis exposure (Harvey, 2012).

In light of the important caveat highlighted above, we felt a critical review of the empirical literature on the cognitive outcomes of children prenatally exposed to cannabis was warranted. In order to assess the clinical significance of findings from the studies reviewed, we determined whether data for cannabis-exposed groups fell outside the average range of functioning when compared against a normative database. If, study investigators did not compare their data with normative scores—this was the case for several studies—we made such comparison ourselves whenever possible. Thus, this article addresses an important gap in our scientific knowledge in that findings should shed light on the extent to which prenatal cannabis exposure produces clinical consequences on offspring. This, of course, could have important public health and policy implications.

## Methods

### Search Strategy

The search strategy employed aimed to identify studies that examined the cognitive effects of prenatal cannabis exposure in humans. Articles up to December 2017 were independently searched by two authors (CAT and CLH) using PsycINFO, PubMed, and Google Scholar. Search terms used keywords: cognitive, pregnancy, and marijuana. Review articles were used as an additional resource to identify studies that might have escaped detection through our initial search. Similarly, reference lists of relevant articles were reviewed for any further potentially eligible studies.

### Inclusion Criteria

The inclusion criteria were: (1) full-text publication in peer-reviewed journal, (2) available in English, (3) assessed cognitive consequences of prenatal cannabis exposure in humans, and (4) provided quantitative measurement of cognitive performance. Studies were excluded if they relied exclusively on questionnaires or brain imaging data as proxies for cognitive functioning.

### Data Extraction

Data was extracted regarding the University and/or research group responsible for conducting the research, cognitive domains tested, participant demographics, cannabis exposure, study findings, and caveats. Additional information was retrieved as to whether individual scores, mean scores, and/or adjusted mean scores were reported, and whether these were compared to normative databases.

#### Clinical Significance Assessment

Cognitive data were assessed for statistically significant differences between children exposed to cannabis prenatally and controls. We then determined whether researchers had compared individual participants' scores against normative databases for the cognitive tasks assessed. When individual scores were not reported, published prenatally exposed group means were compared against the appropriate normative databases, whenever possible. This allowed us to determine whether group means fell outside the normal range of functioning. Norms were obtained from publicly available task administration manuals.

#### Calculating Number of Cognitive Outcomes

The number of cognitive outcomes per study was calculated by summing the number of tasks and/or subtests, and then multiplying by the number of study time-points, prenatal cannabis exposure levels (e.g., light, moderate, heavy), and trimester exposure categories (i.e., first, second, third). This generated the number of statistical comparisons made between different groups, per study. For example, Richardson et al. (1995) used two subtests of the Bayley Scales of Infant Development task (BSID) at two time-points (9 and 19 months) to assess children with light, moderate and heavy prenatal cannabis exposure, with levels of the exposure subdivided by trimester. Therefore, the final calculation is two (task subtests), multiplied by two (study time-points), multiplied by three (exposure levels), multiplied by three (trimesters), for a total of 36 cognitive outcomes assessed. This approach ultimately allowed us to determine the percent of cognitive outcomes on which children who were exposed to cannabis prenatally performed statistically different from controls.

### Rationale for Not Conducting Meta-Analysis

We did not perform a meta-analysis because there were vast differences between studies in quantification of exposure, assessment time-points, location, covariates used, and cognitive outcomes measured. We have instead presented a critical review of the articles embedded within the results section, enabling the reader to be aware of the limitations in interpretation of the prenatal cannabis exposure studies included in this paper. Due to space restrictions, only those studies that reported positive and/or negatively associated cognitive outcomes are critically reviewed in the text of the results section. In the discussion we have additionally provided suggestions for how future studies may improve the reporting, interpretation and communication of their results.

## Review of Results

### Characteristics of Included Studies

**Figure 1:**
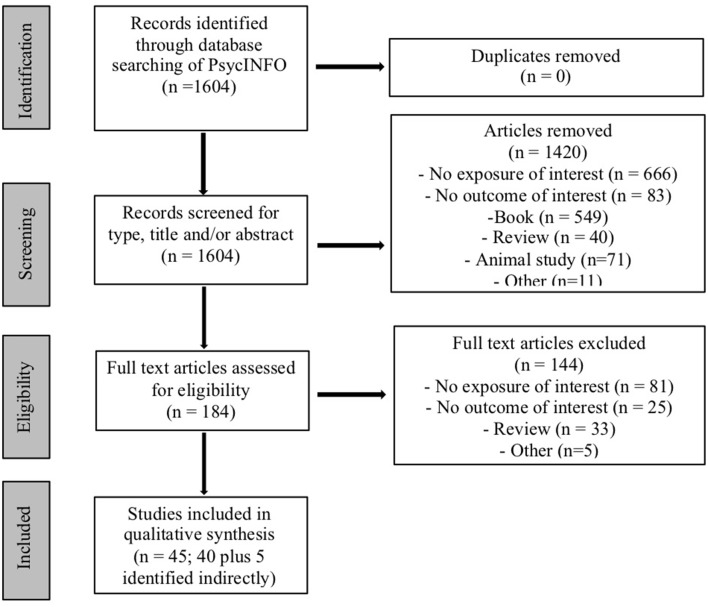
Flowchart of search strategy.

A total of 1,604 articles were identified in the initial search, of which 184 were chosen for full text review after exclusion of irrelevant studies based on title, abstract, keywords, and/or results. Of these, 144 did not meet inclusion criteria and were excluded, with 40 (and 5 additional articles identified indirectly) deemed appropriate for this review based on inclusion criteria (see Figure 1). Individual studies ranged in sample size from 9 (for cannabis-exposed children) to 538 (for control children) participants, and length of follow up ranged from 2 months to 22 years.

The majority of included articles derive from two major longitudinal studies—Ottawa Prenatal Prospective Study (OPPS) and Maternal Health Practices and the Child Development Study (MHPCD). The OPPS examined the potential relationship between prenatal cannabis, tobacco, and alcohol exposure and offspring development in a predominantly White middle-class cohort originally recruited in Ottawa, Canada (Fried and Watkinson, 1988, 1990, 2000, 2001; O'Connell and Fried, 1991; Fried et al., 1992a,b, 1997, 1998, 2003; Smith et al., 2004, 2006, 2016). The second most represented study—MHPCD—focuses primarily on low socioeconomic status African-American women and their offspring recruited from an outpatient prenatal clinic in Pittsburg, Pennsylvania (Day et al., 1994, 2011; Richardson et al., 1995, 2002, 2008, 2009, 2015; Leech et al., 1999; Goldschmidt et al., 2004, 2008, 2012; Willford et al., 2010).

The remaining articles came from seven other studies. The University of Miami's Jamaican Study (UMJS) recruited its participants in Jamaica, and is the only study where participants were recruited outside of Canada and the U.S. (Hayes et al., 1991). Another study we have labeled as the Case Western Reserve University Study (CWRUS), follows a mostly Black cohort located at the county hospital for Cleveland, Ohio (Singer et al., 1999, 2002, 2005, 2008; Noland et al., 2003a,b, 2005; Lewis et al., 2004, 2010). Others we have labeled as the Drexel and Robert Wood Johnson Universities Study (DRWJ), follows children from a predominantly African American sample in the U.S. (Bennett et al., 2008; Carmody et al., 2011) and the Boston and Harvard Universities Study (BHUS), which follows children from a mostly African-American and Caribbean cohort in Boston, Massachusetts (Frank et al., 2005; Beeghly et al., 2006; Rose-Jacobs et al., 2011, 2012). Finally, the remaining articles are from studies conducted by the University of Miami School of Medicine (UMSM) (Morrow et al., 2006), the Yale Child Study Center (YCSC) (Mayes et al., 2007), or the Children's Hospital of Philadelphia (CHP) (Hurt et al., 2005, 2009). Space constraints preclude us from detailing the methodologies in each of these studies; this information can be found in earlier reviews (Day et al., 1985; Fried, 2002; Jaddoe et al., 2012; Huizink, 2014).

### Assessment Tools Used

A total of 30 studies subdivided mothers by whether they consumed cannabis or not during pregnancy (yes/no). Fifteen studies further subdivided participants by self-reported level (“low,” “moderate,” and “heavy”) of cannabis exposure.

Only a third of the studies confirmed the presence of cannabis with biological assays using urine and/or meconium. In addition, few studies (12 out of 45 studies) tested for the potential effects of prenatal cannabis exposure as a function of trimester (first, second, or third) (Table 1).

**Table 1:** Studies assessing cognition in infants and toddlers (up to 24 months).

**Investigators**	**Cohort**	**Domain tested**	**Participants**	**Exposure**	**Findings**	**Caveats**
Fried and Watkinson, 1988	OPPS	*Overall development* {BSID, Mental and psychomotor development index, and Infant behavior record [(Primary cognition composite score composed of object orientation, goal directedness, attention span, reactivity, and vocalization subtests), (Auditory score composed of responsiveness to persons, examiner and mother, reactivity, and listening to sounds subtests), (Visual score composed of responsiveness to objects, attention span, manipulating, and sights-looking subtests)]} **24 months only:** *Language development* [Reynell developmental languages scales, (Comprehension and expression subtests)] **Total no. of outcome measures** **=** 41	12 and 24 month children of women who reported MJ use during pregnancy **12 month olds:** (heavy MJ: *N* = 17; CTL: *N* = 162) **24 month olds:** (heavy MJ: *N* = 18; CTL: *N* = 100)	MJ use self-reported throughout for each trimester **Categories:** Exposed Heavy = AWJ > 5	**12 month olds:** Prenatal MJ exposure was associated with higher cognition scores (BSID: primary cognition composite)^†^ No other differences were observed	Relatively small number of participants studied in the heavy MJ-exposed group Mothers reported tobacco cigarette smoking, and alcohol use. It is not clear whether this was controlled for Maternal MJ use was determined exclusively from self-report
Richardson et al., 1995	MHPCD	*Overall development* (BSID, Mental and psychomotor development index) **Total no. of outcome measures** **=** 36	9 and 19 month children of women who reported MJ use during pregnancy **9 month olds:** (heavy MJ: *N* = 73; moderate MJ: *N* = 31; light MJ: *N* = 104; CTL: *N* = 312) **19 month olds:** (heavy MJ: *N* = 57; moderate MJ: *N* = 34; light MJ: *N* = 119; CTL: *N* = 358)	MJ use self-reported at 4th & 7th pregnancy months and at 24–28 h post-delivery for each trimester **Categories:** Exposed by trimester Light = 0 < ADJ < 0.4 Moderate = 0.4 ≤ ADJ < 1 Heavy = ADJ ≥ 1	**9 month olds/3rd trimester:** Heavy prenatal MJ exposure group performed more poorly on cognitive development (BSID: mental development index)^*^ Prenatal MJ exposure was associated with lower cognition scores (BSID: mental development index)^*^ No other differences were observed	When adjusted mean cognitive scores of all groups studied were compared against a normative data set by the authors, they exceeded the average range Mothers reported tobacco cigarette smoking, and alcohol use, but this was controlled for Maternal MJ use was determined exclusively from self-report
Singer et al., 1999	CWRUS	*Visual recognition memory* (Fagan test of infant intelligence, novelty score for four problems) **Total no. of outcome measures** **=** 4	Up to 2 month children of women who self-reported MJ use during pregnancy: (MJ: *N* = 58; CTL = 16)	MJ use self-reported at “as soon as possible” post-delivery period Also, maternal and infant urine analyzed for cannabinoids; infant meconium was analyzed for THC **Categories:** Exposed	No differences were observed	Cognitive scores were not reported Mothers reported other drug use. It is not clear whether this was controlled for
Singer et al., 2002	CWRUS	*Overall development* (BSID-II, Mental and psychomotor development index) **Total no. of outcome measures** **=** 6	6, 12, and 24 month children of women who reported MJ use during pregnancy: (MJ: *N* = 102; CTL = 313)	MJ use self-reported at “as soon as possible” post-delivery period Also, maternal and infant (in some cases) urine analyzed for cannabinoids; infant	No differences were observed	Cognitive scores were not reported Mothers reported tobacco cigarette smoking, alcohol, and cocaine use, but this was controlled for meconium was analyzed for cannabinoids **Categories:** Exposed
Noland et al., 2003a	CWRUS	*Goal-directed action* (A-not-B task); *Overall development* (BSID-II, Mental and psychomotor development index, and behavioral rating scale) **Total no. of outcome measures** **=** 4	9 to 12 month children of women who reported MJ use during pregnancy: (MJ: *N* = 9; CTL: *N* = 42)	MJ use self-reported 2 weeks post-delivery Also, maternal and infant urine analyzed for THC **Categories:** Exposed	No differences were observed	Cognitive scores were not reported Mothers reported tobacco cigarette smoking, alcohol, and cocaine use, but this was controlled for Relatively small number of participants studied in the MJ-exposed group
Singer et al., 2005	CWRUS	*Visual recognition memory* (Fagan test of infant intelligence, novelty score, percent performing in risk range, familiarization phase average looking time, and trial phase average looking time) **Total no. of outcome measures** **=** 24	6 and 12 month children of women who reported MJ use during pregnancy: (MJ: *N* = 107; CTL: *N* = 245)	MJ use self-reported 2 weeks post-delivery Also, maternal and infant urine analyzed for THC **Categories:** Exposed by trimester	**6 month olds:** Severity of prenatal MJ exposure in the 3rd trimester was associated with shorter average looking times^†^ No other differences were observed	Faster looking times did not relate to visual recognition memory Mothers reported tobacco cigarette smoking, alcohol, and benzodiazepine use, but this was controlled for
Richardson et al., 2008	MHPCD	*Overall development* {BSID, Mental and psychomotor development index, and Infant behavior record [(Primary cognition composite score composed of object orientation, goal directedness, attention span, reactivity and vocalization subtests), (Auditory score composed of responsiveness to persons, examiner and mother, reactivity, and listening to sounds subtests), (Visual score composed of responsiveness to objects, attention span, manipulating, and sights-looking subtests)]} **Total no. of outcome measures** = 57	16 month children of women who reported MJ use during pregnancy: (MJ: *N* = 85; CTL: *N* = 176)	MJ use self-reported at 7th pregnancy month and at 24 h post-delivery for each trimester **Categories:** Exposed by trimester	No differences were observed	Cognitive scores were not reported Mothers reported tobacco cigarette smoking, and alcohol use, but this was controlled for Maternal MJ use was determined exclusively from self-report

*ADJ, Average daily joints; AWJ, Average weekly joints; BSID/BSID-II, Bayley scales of infant development 1st and 2nd editions; CTL, Control group; CWRUS, Case Western Reserve University Study; MHPCD, Maternal Health Practices and the Child Development Study; MJ, Marijuana group; NIAAA, National Institute on Alcohol Abuse and Alcoholism; NIDA, National Institute on Drug Abuse; OPPS, Ottawa Prenatal Prospective Study; THC, Tetrahydrocannabinol*.

* *Negative associations on cognitive outcomes*,

† *Positive associations on cognitive outcomes*.

The cognitive domains tested, participant demographics, level of cannabis exposure, proposed findings, and caveats for all included studies are presented in Tables 1–4. Due to the different types of exposure categorization and cognitive measures used across studies, results were grouped by age at which the prenatally exposed children were assessed. Seven studies reported on infants and toddlers (up to 24 months); 19 reported on children (3 to 9 years); 12 reported on early adolescence (9 to 12 years), and eight reported on adolescence and early adulthood (13 to 22 years).

Tables A1–A4 in Supplementary Material outline whether each study reported individual, group mean, and adjusted group mean cognitive task scores and compared them to normative data. When cognitive task scores were reported, and normative databases were publicly available, we carried out the comparisons ourselves and report the results below.

### Summary of Results

In general, prenatal cannabis exposure was associated with few effects, negative or positive. Of the 1,004 cognitive outcomes assessed, children with prenatal cannabis exposure performed more poorly on 34 (3.4%) and better on 9 (0.9%) when compared to a control group.

Cognitive task scores for *individual* participants were not included or compared to normative data in any of the studies reviewed. Mean cognitive task scores were reported in 17 studies and compared against normative data by the authors on only one occasions (Rose-Jacobs et al., 2011). Adjusted group means were reported in seven studies and compared to normative data by the authors on two occasions (Richardson et al., 1995; Rose-Jacobs et al., 2011). Because the performance of children exposed to cannabis prenatally did not differ from non-exposed children on the majority of cognitive outcomes, we will discuss the studies where significant effects were found.

#### Studies Assessing Infants and Toddlers (0–24 Months)

Table 1 shows studies examining the association between prenatal cannabis exposure and cognitive functioning in infants and toddlers. A total of 169 cognitive outcomes were assessed. About half (55 percent) were obtained from the MHPCD, 22 percent from the OPPS, and 22 percent from CWRUS.

Generally, prenatal cannabis exposure was not associated with cognitive performance. However, prenatal cannabis exposure was associated with worse performance on two cognitive outcomes (Richardson et al., 1995) and better performance on two cognitive outcomes (Fried and Watkinson, 1988; Singer et al., 2005). Cognitive performance scores were compared to a normative database by the authors in one (Richardson et al., 1995) of the seven articles (Table A1 in Supplementary Material).

Fried and Watkinson (1988) assessed the cognitive functioning of 12- and 24-month old children whose mothers reported using over 5 joints per week during their pregnancies. Of 38 cognitive outcomes reported, one significant association was found. Children who were prenatally exposed to cannabis performed *better* than non-exposed children on the BSID as indicated by higher Primary Composite scores. It is important to point out that although unadjusted mean scores were reported; they were not compared to normative data. We attempted to obtain normative scores but were unable to do so, making it difficult to determine the clinical relevance of the finding. It is also important to note that there was a relatively small number (*N* = 17) of children in the group with prenatal cannabis exposure.

Singer et al. (2005) studied a considerably larger sample (*N* = 107) of infants with prenatal cannabis exposure. Of the 24 cognitive outcomes assessed, one significant positive association was found: prenatal cannabis exposure in the third trimester was associated with shorter average looking times on the Visual Recognition Memory task. However, shorter average looking times were not associated with visual recognition memory. Task scores were not reported, nor were they compared against a normative dataset making it difficult to determine the clinical relevance of the finding.

Unlike the study above, Richardson et al. (1995) compared cognitive task scores to normative data. Of the 36 cognitive outcomes assessed, two significant associations were reported. Third trimester marijuana use predicted lower scores on the BSID mental development index in 9-month old infants. Additionally, infants who were prenatally exposed to more than one joint per day in the third trimester performed more poorly than controls on the BSID mental development index. Importantly, both group's scores on this task were above the normal range when adjusted to account for confounding variables (Table A1 in Supplementary Material).

#### Studies Assessing Young Children (3–9 Years)

Table 2 shows studies examining the association between prenatal cannabis exposure and cognitive functioning in children aged 3 to 9 years. A total of 397 cognitive outcomes were assessed. The largest proportion of cognitive outcomes (46 percent) derived from the MHPCD. The OPPS contributed 27 percent, the UMJS 9 percent, and the CWRUS 8 percent. Another 5 percent came from the DRWJ. Finally, the remaining 10 percent came either from the BHUS, UMSM or the YCSC studies.

**Table 2:** Studies assessing cognition in children (3 to 9 years).

**Investigators**	**Cohort**	**Domain tested**	**Participants**	**Exposure**	**Findings**	**Caveats**
Fried and Watkinson, 1990	OPPS	*Overall development* [McCarthy's Scales of Children's Abilities, (General cognitive index composed of verbal, perceptual and quantitative subtests), and memory and motor subtests]; *Language development* [Reynell developmental languages scales, (Comprehension and expression subtests)] **48 month only:** *Stereognosis* (Tactile form recognition task); *Visuomotor coordination* (Pegboard test); *Auditory comprehension/processing* (PPVT-R form L, vocabulary) **Total no. of outcome measures** **=** 38	3 and 4 year old children of women who reported MJ use during pregnancy **3 year olds:** (heavy MJ: *N* = 19; moderate MJ: *N* = 12; infrequent-no use: *N* = 102) **4 year olds:** (heavy MJ: *N* = 19; moderate MJ: *N* = 10; infrequent-no use: *N* = 101)	MJ use self-reported throughout pregnancy **Categories:** Exposed Infrequent-no use = AWJ < 1 Moderate = 1 ≤ AWJ < 6 Heavy = AWJ ≥ 6	**3 year olds:** Moderate prenatal MJ exposure was associated with better motor performance (McCarthy's Scales)^†^ **4 year olds:** Heavy prenatal MJ exposure was associated with poorer verbal^*^ and memory^*^ performance (McCarthy's Scales) and auditory comprehension/ processing (PPVT-R form L, vocabulary)^*^ No other differences were observed	Verbal and auditory comprehension/ processing performance: Adjusted cognitive data not reported nor compared against normative data set. Thus, the clinical importance of findings could not be determined Memory performance: Although adjusted cognitive data was reported, data was not compared against a normative data set Participants in MJ exposure group were not compared to an appropriate control group (AWJ = 0) Mothers who reported heavy MJ use had lower levels of education, were younger and provided a poorer home environment, as measured by the HOME, than other groups, but this was controlled for Relatively small number of participants studied in the MJ-exposed group Maternal MJ use was determined exclusively from self-report
Hayes et al., 1991	UMJS	*Overall development* [McCarthy's Scales of Children's Abilities, (General cognitive index composed of verbal, perceptual, and quantitative subtests), and memory and motor subtests] **Total no. of outcome measures** **=** 36	4 and 5 year old children of women who reported MJ use during pregnancy: (MJ: *N* = 30; CTL *N* = 26)	Not explicitly stated, but apparently self-reported MJ use **Categories:** Light = AWJ < 10 Moderate = 11 ≤ AWJ ≤ 20 Heavy = 21 ≤ AWJ ≤ 70	No differences were observed	Cognitive scores were not reported Authors mention study not confounded by “polydrug use, alcohol consumption, tobacco smoking…,” but other drug use was not reported Maternal MJ use was determined exclusively from self-report
O'Connell and Fried, 1991	OPPS	General intelligence [WISC-R, (full scale, verbal and performance IQ, verbal comprehension, perceptual organization and freedom from distractibility subtests)]; *Basic visuoperceptual functioning* (TVPS, perceptual quotient and visual discrimination, memory, spatial relationships, form constancy, sequential memory, figure-ground and closing subtests); *Visuomotor coordination* (DTVMI, Draw a man test); *Visual attention/task shifting* (Trail making test part A); *Motor development* (Finger-tapping test); *Visual attention span/memory/sequencing* (Knox cube; Stroop color and word	6 to 9 year old children of women who reported MJ use during pregnancy: (MJ: *N* = 28; CTL: *N* = 28)	MJ use self-reported throughout pregnancy **Categories:** Exposed	No differences were observed	Mothers reported tobacco cigarette smoking, and alcohol use, but this was controlled for Maternal MJ use was determined exclusively from self-report test); *Language comprehension* [Test of language development-primary, (Syntax quotient composed of grammatical understanding, sentence imitation, and grammatical completion subtests)]; *Academic achievement* (WRAT-R, reading recognition, spelling and arithmetic subtests; Woodcock reading mastery test, passage comprehension subtests) **Total no. of outcome measures** **=** 27
Fried et al., 1992a	OPPS	*Impulsivity/sustained attention* (Gordon delay task, rewards, responses and efficiency ratio; Gordon vigilance task, total correct, omissions, commissions); *Memory* (McCarthy's Scales of Children's Abilities, memory subtest) **Total no. of outcome measures** **=** 14	6 year old children of women who reported MJ use during pregnancy: (heavy MJ: *N* = 19; moderate MJ: *N* = 14; infrequent-no use: *N* = 93)	MJ use self-reported throughout pregnancy **Categories:** Infrequent-no use = AWJ < 1 Moderate = 1 ≤ AWJ < 6 Heavy = AWJ ≥ 6	Severity of prenatal MJ use was associated with poorer performance on a measure of impulsivity/sustained attention (Gordon vigilance task, total correct and omissions)^*^ No other differences were observed	Cognitive data not reported nor compared against normative data set. Thus, the clinical importance of findings could not be determined. All mean performance scores did not differ as a function of group. Mothers reported tobacco cigarette smoking, and alcohol use, which makes it difficult to assess the effect of MJ Participants in MJ exposure group were not compared to an appropriate control group (AWJ = 0) Mothers who reported heavy MJ use had lower levels of education than other groups, but this was controlled for Relatively small number of participants studied in MJ-exposed groups Maternal MJ use was determined exclusively from self-report
Fried et al., 1992b	OPPS	*Overall development* [McCarthy's Scales of Children's Abilities, (General cognitive index composed of verbal, perceptual, and quantitative subtests), and memory and motor subtests]; *Auditory comprehension/processing* (PPVT-R form L) **Total no. of outcome measures** **=** **28**	5 and 6 year old children of women who reported MJ use during pregnancy **5 year olds:** (heavy MJ: *N* = 21; moderate MJ: *N* = 11; infrequent-no use: *N* = 103) **6 year olds:** (heavy MJ: *N* = 22; moderate MJ: *N* = 14; infrequent-no use: *N* = 101)	MJ use self-reported throughout pregnancy **Categories:** Infrequent-no use = AWJ < 1 Moderate = 1 ≤ AWJ < 6 Heavy = AWJ ≥ 6	No differences were observed	Mothers reported tobacco cigarette smoking, and alcohol use, which makes it difficult to assess the effect of MJ Participants in MJ exposure group were not compared to an appropriate control group (AWJ = 0) Mothers who reported heavy MJ use had lower levels of education than other groups, which makes it difficult to assess the effect of MJ Relatively small number of participants studied in MJ-exposed groups Maternal MJ use was determined exclusively from self-report
Day et al., 1994	MHPCD	*General intelligence* [SBIS-IV, (Composite score composed of verbal, quantitative and abstract/visual reasoning, and short-term memory subtests)] **Total no. of outcome measures** **=** 135	3 year old children of women who reported MJ use during: 1st trimester (heavy MJ: *N* = 89; moderate MJ: *N* = 48; light MJ: *N* = 130; CTL: *N* = 386), 2nd trimester (heavy MJ: *N* = 28; moderate MJ: *N* = 21; light MJ: *N* = 79; CTL: *N* = 466), 3rd trimester (heavy MJ: *N* = 28; moderate MJ: *N* = 23; light MJ: *N* = 66; CTL: *N* = 538)	MJ use self-reported at 4th & 7th pregnancy months and at 24–28 h post-delivery **Categories:** Light = 0 < ADJ ≤ 0.4 Moderate = 0.4 < ADJ < 1 Heavy = ADJ ≥ 1	**1st trimester:** Among children with African-American mothers, severity of MJ exposure was associated with lower general intelligence scores (SBIS-IV: verbal reasoning)^*^ **2nd trimester:** Among children with African-American mothers, severity of MJ exposure was associated with lower general intelligence scores (SBIS-IV: short-term memory)^*^ No other differences were observed	Cognitive scores were not reported or compared against a normative data set. Thus, the clinical importance of findings could not be determined Only one cognitive measure used to assess a specific domain Overall, mothers who reported heavy MJ use had higher rate of alcohol and illicit drug use than CTL mothers. It is not clear whether this was controlled for in the African-American sub-sample African-American mothers who reported MJ use had lower levels of education than African-American CTL mothers Maternal MJ use was determined exclusively from self-report
Leech et al., 1999	MHPCD	*Attention/vigilance* (CPT-III); *General intelligence* (SBIS-IV, Composite score) **Total no. of outcome measures** **=** 6	6 year old children of women who reported MJ use during: 1st trimester (heavy MJ: *N* = 90; moderate MJ: *N* = 48; light MJ: *N* = 110; CTL: *N* = 360), 2nd trimester (heavy MJ: *N* = 31; moderate MJ: *N* = 23, light MJ: *N* = 84; CTL *N* = 471), 3rd trimester (heavy MJ: *N* = 31; moderate MJ: *N* = 20; light MJ: *N* = 64; CTL *N* = 493)	MJ use self-reported at 4th & 7th pregnancy months and at 24–28 h post-delivery **Categories:** Exposed by trimester	**2nd trimester:** Prenatal MJ exposure was associated with poorer (more errors of commission)^*^ and better (fewer errors of omission) CPT-III performance^†^ No other differences were observed	Cognitive scores were not reported or compared against a normative data set. Thus, the clinical importance of findings could not be determined SBIS-IV data not reported Only one cognitive measure used to assess a specific domain Mothers reported tobacco cigarette smoking, alcohol, and cocaine use, which makes it difficult to assess the effect of MJ Maternal MJ use was determined exclusively from self-report
Noland et al., 2003b	CWRUS	*Impulse control* (Tapping inhibition task adaptation); *Verbal fluency* (McCarthy's Scales of Children's Abilities, category fluency subtest adaptation); *Motor coordination* (Motor-planning task adaptation) **Total no. of outcome measures** **=** 3	4 year old children of women who reported MJ use during pregnancy (MJ: *N* = 53–76; CTL *N* = 116–194)	MJ use self-reported 2 weeks post-delivery Also, maternal and infant urine analyzed for THC **Categories:** Exposed	No differences were observed	Average maternal verbal IQ score was below the normal range (75.1) Tapping inhibition task adjusted cognitive scores were not reported McCarthy's Scales of Children's Abilities cognitive scores were not reported Only one cognitive measure used to assess a specific domain Mothers reported tobacco cigarette smoking, alcohol, cocaine, and benzodiazepine use, which makes it difficult to assess the effect of MJ
Lewis et al., 2004	CWRUS	*Language development* (CELF-P, receptive, expressive and total language, linguistic and basic concepts, sentence structure, recalling sentences, formulating labels and word structure) **Total no. of outcome measures** **=** 9	4 year old children of women who reported MJ use during pregnancy (MJ: *N* = unknown; CTL *N* = unknown)	MJ use self-reported immediately after delivery Also, maternal urine and infant meconium analyzed for THC **Categories:** Exposed	Prenatal MJ exposure group performed more poorly on measure of formulating labels (CELF-P)^*^ No other differences were observed	Cognitive data not reported nor compared against normative data set. Thus, the clinical importance of findings could not be determined Only one cognitive measure used to assess a specific domain Mothers reported tobacco cigarette smoking, alcohol, and cocaine use, and this was not controlled for Unknown number of participants in MJ and CTL groups
Frank et al., 2005	BHUS	*General intelligence* (WPPSI-R, full scale IQ, verbal IQ, and performance IQ) **Total no. of outcome measures** **=** 3	48 month children of women who reported MJ use during pregnancy: (MJ: *N* = 38; CTL: *N* = 170)	MJ use self-reported at post-delivery period Also, maternal and infant urine analyzed for cannabinoids; infant meconium was analyzed for cannabinoids **Categories:** Exposed	No differences were observed	Cognitive scores were not reported Only one cognitive measure used to assess a specific domain Mothers reported tobacco cigarette smoking, alcohol, and cocaine use, which makes it difficult to assess the effect of MJ
Noland et al., 2005	CWRUS	*Attention/vigilance* (CPT); *Selective attention* (PDT) **Total no. of outcome measures** **=** 2	4 year old children of women who reported MJ use during pregnancy (MJ: *N* = 85; CTL *N* = 216)	MJ use self-reported 2 weeks post-delivery Also, maternal and infant urine analyzed for THC **Categories:** Exposed	No differences were observed	Cognitive scores were not reported or compared against a normative data set. Thus, the clinical importance of findings could not be determined Mothers reported tobacco cigarette smoking, alcohol, and cocaine use, which makes it difficult to assess the effect of MJ
Beeghly et al., 2006	BHUS	**6 years only:** *Language development* (TOLD-P3, receptive, expressive and total language) **9 years only:** *Language development* (CELF-3, receptive, expressive and total language) **Total no. of outcome measures** **=** 6	6 and 9 year old children of women who reported MJ use during pregnancy: (MJ: *N* = 35; CTL *N* = 125)	MJ use self-reported at post-delivery period Also, maternal and infant urine analyzed for cannabinoids; infant meconium was analyzed for cannabinoids **Categories:** Exposed	No differences were observed	Cognitive scores were not reported Mothers reported tobacco cigarette smoking, alcohol, and cocaine use, which makes it difficult to assess the effect of MJ
Morrow et al., 2006	UMSM	*Academic achievement* (WIAT screener, Mathematics and screener composite scores and basic reading, spelling, mathematics reasoning and numerical operations subtests); *General intelligence* (WISC-III short form, full scale IQ) **Total no. of outcome measures** **=** 7	Seven year old children of women who reported MJ use during pregnancy: (MJ: *N* = unknown; CTL *N* = unknown)	MJ use self-reported within 36 h post-delivery Also, maternal and infant urine analyzed for cannabinoids; infant meconium was analyzed for cannabinoids **Categories:** Exposed	No data was reported	Although children were assessed, cognitive scores nor a comparison between MJ and CTL groups were reported Study population WISC-III and WIAT test scores were below the normal range (IQ < 80). Thus, even if test scores for MJ and CTL groups would had been compared, the clinical importance of findings could not have been determined Unknown number of children in each group
Mayes et al., 2007	YCSC	*Visuospatial immediate/short-term memory* (Groton maze learning test, total correct moves per second, and number of errors) **Total no. of outcome measures** **=** 2	8 to 10 year old children of women who reported MJ use during pregnancy: (MJ: *N* = 41; CTL *N* = 89)	MJ use in last 30 days self-reported prenatally and/or at post-delivery period Also, maternal urine was analyzed for THC **Categories:** Exposed	No differences were observed	Cognitive scores were not reported Only one cognitive measure used to assess a specific domain Although the cognitive test is computerized, participants might have received different levels of performance-related coaching by experimenters Mothers reported tobacco cigarette smoking, alcohol, and cocaine use Participants were recruited at different time points during pregnancy
Bennett et al., 2008	DRWJ	*General intelligence* [SBIS-IV short form, (Composite IQ score composed of short-term memory, and abstract/visual, quantitative and verbal reasoning subtests)] **Total no. of outcome measures** **=** 15	4, 6, and 9 year old children of women who reported MJ use during pregnancy: (MJ: *N* = 35; CTL *N* = 196)	MJ use self-reported prenatally and/or at post-delivery period **Categories:** Exposed	No differences were observed	Cognitive scores were not reported Only one cognitive measure used to assess a specific domain Mothers reported tobacco cigarette smoking, alcohol, and cocaine use, but this was controlled for Relatively small number of participants studied in the MJ-exposed group Participants were recruited at different time points during pregnancy
Goldschmidt et al., 2008	MHPCD	*General intelligence* [SBIS-IV, (Composite score composed of verbal reasoning, quantitative reasoning, abstract/visual reasoning, and short-term memory subtests)] **Total no. of outcome measures** **=** 30	6 year old children of women who reported MJ use during: 1st trimester (heavy MJ: *N* = 93; light-moderate MJ: *N* = 175; CTL: *N* = 380), 2nd trimester (heavy MJ: *N* = 30; light-moderate MJ: *N* = 103; CTL *N* = 455); 3rd trimester (heavy MJ: *N* = 32; light-moderate MJ: *N* = 88; CTL *N* = 528)	MJ use self-reported at 4th & 7th pregnancy months and at 24–28 h post-delivery **Categories:** Light-moderate = 0 < ADJ < 1 Heavy = ADJ ≥ 1	**1st trimester:** Heavy prenatal MJ exposure group performed more poorly on measure of verbal reasoning^*^ **2nd trimester:** Heavy prenatal MJ exposure group performed more poorly on measures of short-term memory, ^*^ quantitative reasoning, ^*^ and the composite score^*^ **3rd trimester:** Heavy prenatal MJ exposure group performed more poorly on measure of quantitative reasoning^*^ No other differences were observed	Adjusted cognitive scores were not reported or compared against a normative data set. Thus, the clinical importance of findings could not be determined Participants performed in the normal range on the majority of the cognitive tests. There were three exceptions using unadjusted scores: (1) 2nd trimester exposure - composite score (1 point below the norm); (2) 2nd trimester exposure quantitative reasoning score (2 points below); and (3) 3rd trimester exposure - quantitative reasoning score (1 point below) Only one cognitive measure used to assess a specific domain Mothers who used MJ during pregnancy were also more likely to report using alcohol, tobacco, and cocaine, which makes it difficult to assess the effect of MJ Mothers who used MJ during pregnancy were more likely to be poor, single, and provide a poorer home environment, as measured by the HSQ, which makes it difficult to assess the effect of MJ Relatively small number of participants studied in heavy MJ-exposed group Maternal MJ use was determined exclusively from self-report
Singer et al., 2008	CWRUS	*General intelligence* (WISC-IV, full scale IQ, and verbal comprehension, perceptual reasoning, processing speed, and working memory subtests); *Academic achievement* (Woodcock Johnson-III Tests of Achievement) **Total no. of outcome measures** **=** 18	9 year old children of women who reported MJ use during pregnancy: (MJ: *N* = 114; CTL *N* = 257)	MJ use self-reported at post-delivery period for previous month and each trimester Also, maternal and infant urine was analyzed for cannabinoids **Categories:** Exposed by trimester	**3rd trimester:** Prenatal MJ exposure group performed more poorly on measure of processing speed (WISC-IV, coding)^*^ No other differences were observed	Cognitive scores were not reported or compared against a normative data set. Thus, the clinical importance of findings could not be determined Mothers reported tobacco cigarette smoking, alcohol, and cocaine use, and exposure to lead, which makes it difficult to assess the effect of MJ
Richardson et al., 2009	MHPCD	*General intelligence* [SBIS-IV, (Composite score composed of verbal reasoning, abstract/visual reasoning, and short-term memory subtests)] **Total no. of outcome measures** **=** 12	3 year old children of women who reported MJ use during pregnancy: (MJ: *N* = 56; CTL *N* = 200)	MJ use self-reported at 7th pregnancy months and/or at 24 h post-delivery **Categories:** Exposed by trimester	**1st trimester:** Prenatal MJ exposure group performed more poorly on measure of general intelligence^*^ (abstract/visual reasoning and composite scores)^*^ No other differences were observed	Cognitive scores were not reported or compared against a normative data set. Thus, the clinical importance of findings could not be determined Mothers reported tobacco cigarette smoking, alcohol, and cocaine use, which makes it difficult to assess the effect of MJ Maternal MJ use was determined exclusively from self-report
Carmody et al., 2011	DRWJ	*Attention and inhibitory control* (Yale child study center attention task, overall accuracy score, inhibition errors and attention errors) **Total no. of outcome measures** **=** 6	6 year old children of women who reported MJ use during pregnancy (MJ: *N* = unknown; CTL: *N* = unknown) Also, infant meconium was analyzed for THC	MJ use self-reported at either prenatally, at post-delivery period or in mother's home within 2 weeks of child's birth **Categories:** Exposed or exposed by gender	No differences were observed	Cognitive data not reported Mothers reported tobacco cigarette smoking, alcohol and cocaine use, which makes it difficult to assess the effect of MJ Unknown number of children in each group

*ADJ, Average daily joints, AWJ, Average weekly joints; BHUS, Boston and Harvard Universities Study; CELF-3, Child evaluation of language fundamentals-third edition; CELF-P, Clinical evaluation of language fundamentals-preschool 1st edition; CPT/CPT-III, Continuous performance test versions I and III; CTL, Control group; CWRUS, Case Western Reserve University Study; DRWJ, Drexel and Robert Wood Johnson Universities Study; DTVMI, Developmental test of visual-motor integration; MHPCD, Maternal Health Practices and the Child Development Study; MJ, Marijuana group; NCRR, National Center for Research Resources; NIAAA, National Institute on Alcohol Abuse and Alcoholism; NIDA, National Institute on Drug Abuse; OPPS, Ottawa Prenatal Prospective Study; PDT; Picture deletion task for preschoolers-modified; SBIS-IV, Stanford-Binet intelligence scale 4th edition; THC, Tetrahydrocannabinol; TOLD-P3, Test of language development-primary third edition; TVPS, Test of visual-perceptual skills; UMJS, University of Miami's Jamaican Study; UMSM, University of Miami School of Medicine Study; WIAT, Wechsler individual achievement test 1st edition; WISC-III/WISC-IV/WISC-R, Wechsler intelligence scale for children 3rd, 4th, and revised editions; WPPSI-R, Wechsler preschool and primary scales of intelligence-revised; WRAT-R, Wide range achievement test-revised; YCSC, Yale Child Study Center*.

* *Negative associations on cognitive outcomes*,

† *Positive associations on cognitive outcomes*.

Performance on the vast majority (95.7%) of the 380 cognitive outcomes assessed was similar between the groups. Prenatal cannabis exposure was significantly associated with 17 cognitive outcomes: better performance on two (Fried and Watkinson, 1990; Leech et al., 1999) and worse performance on 15 (Fried and Watkinson, 1990; Day et al., 1994; Leech et al., 1999; Lewis et al., 2004; Goldschmidt et al., 2008; Singer et al., 2008; Richardson et al., 2009). For this age group, no group of investigators compared cognitive task scores to a normative database (Table A2 in Supplementary Material). In addition, cognitive scores were not reported for the majority of studies (68%) (Hayes et al., 1991; Day et al., 1994; Leech et al., 1999; Lewis et al., 2004; Frank et al., 2005; Noland et al., 2005; Beeghly et al., 2006; Morrow et al., 2006; Mayes et al., 2007; Bennett et al., 2008; Singer et al., 2008; Richardson et al., 2009; Carmody et al., 2011).

Leech et al. (1999) assessed the cognitive functioning of 6-year old children whose mothers reported cannabis during pregnancy (maximum *N* = 110). Of the 6 cognitive outcomes assessed, two significant associations were found. Prenatal cannabis exposure was associated with both *better* (fewer omission errors) and poorer (more commission errors) on the Continuous Performance Test—Version III (CPT-III). However, the authors did not report or compare scores to those from a normative database.

These mixed findings appear to conflict with those from a previous study. Day et al. (1994), assessed the cognitive functioning of 3-year old children with prenatal cannabis exposure (maximum *N* = 110). Of the 135 cognitive outcomes assessed, two significant associations were found. Among children with African-American mothers, severity of cannabis exposure during the first and second trimesters was associated with lower scores on the SBIS-IV verbal reasoning and short-term memory subscales, respectively. However, the authors did not report or compare scores to those from a normative database, again making it difficult to determine the clinical significance of the findings. Furthermore, mothers who reported heavy cannabis use also had higher rates of alcohol and illicit drug use than controls. It is unclear whether this was controlled for in the African-American subsample, which makes it difficult to isolate the potential effects of prenatal cannabis exposure from those of other drugs.

Similarly, Lewis et al. (2004), did not control for other drug use in their study of language development in 4-year old children with prenatal cannabis exposure (*N* = unknown). Of the nine cognitive outcomes assessed, one significant association was found. Children in the prenatal cannabis exposure group performed more poorly on the CELF-P formulating labels task. However, the authors did not report or compare scores to those from a normative database, so the clinical significance of the finding is difficult to determine.

In an attempt to minimize the impact of other drug use, Singer et al. (2008), conducted a study in which the cognitive functioning of 9-year-old children with prenatal cannabis exposure was examined (*N* = 114). In addition, these researchers controlled for mothers' cigarette smoking, alcohol use and cocaine use (Singer et al., 2008). Of the 18 cognitive outcomes assessed, one significant association was found. Prenatal cannabis exposure in the third trimester was associated with poor performance on a WISC-IV measure of processing speed. However, the investigators did not report individual or group scores, nor did they compare scores against a normative database.

In a study conducted by Richardson et al. (2009), the cognitive functioning of 3-year-old children who were exposed to cannabis prenatally was assessed (*N* = 56), and the researchers also controlled for mothers' use of other drugs. Of the 12 cognitive outcomes assessed, two significant associations were found. Children with prenatal cannabis exposure during the first trimester performed more poorly on the SBIS-IV, as evidenced by lower abstract/visual reasoning and composite scores. Again, the researchers did not report or compare scores to those from a normative database.

Fried and Watkinson (1990) conducted a study with 3 and 4-year-old children of women who reported cannabis use during pregnancy (maximum *N* = 19). Of the 38 cognitive outcomes assessed, 4 significant associations were found. For the 3-year-old children, prenatal cannabis exposure of 1 to 6 average weekly joints was associated with better motor performance on the McCarthy's Scales of Children's Abilities motor subtest. On the other hand, for 4-year-old children, exposure of over 6 average weekly joints was associated with poorer performance on the verbal and memory subtests, and the PPVT-R. Unlike the above studies, Fried and Watkinson (1990) reported mean cognitive task scores for the study groups. However, normative data for the tasks were not publicly available, which prevented us from comparing the mean scores to normative data ourselves. Therefore, the clinical significance of the study findings could not be determined.

In a similar study, Fried et al. (1992a) assessed the cognitive functioning of 6-year old children with prenatal cannabis exposure (maximum *N* = 19). Of the 14 cognitive outcomes assessed, one significant association was found. Prenatal cannabis use was associated with poorer performance on the Gordon Vigilance task. Although unadjusted mean task scores were reported, the authors did not compare them (or adjusted scores) against normative data.

Finally, for the study by Goldschmidt et al. (2008), we were able assess the clinical relevance of the study findings. Out of 30 cognitive outcomes assessed, five significant associations were found. Children with prenatal cannabis exposure (maximum *N* = 175) of more than one joint per day in the first and second trimesters performed worse than children of abstainers on five SBIS-IV measures: short-term memory, verbal and quantitative reasoning domains, and composite scores. Although Goldschmidt et al. (2008) reported cognitive task scores for the study groups they did not compare them to normative data. However, because the published norms for the tasks were publicly available, we were able to conduct the comparison ourselves. The composite score in the 2nd trimester exposure group and the quantitative reasoning score for the 3rd trimester exposure group fell 1 point below the normal range. Furthermore, the quantitative reasoning subscale score for the 2nd trimester exposure group fell two points below the normal range (See Table A2 in Supplementary Material). It is important to note that although mean scores fell below the normal range of performance, the task scores were unadjusted for covariates.

#### Studies Assessing Early Adolescents (9–12 Years)

Table 3 shows studies assessing the association between prenatal cannabis exposure and cognitive performance in early adolescents. A total of 278 cognitive outcomes were assessed. The majority (55 percent) were obtained from the OPPS. The MHPCD followed with 27 percent. Two BHUS articles yielded seven percent and the remaining 11 percent came from three other study cohorts.

**Table 3:** Studies assessing cognition in early adolescence (9 to 12 years).

**Investigators**	**Cohort**	**Domain tested**	**Participants**	**Exposure**	**Findings**	**Caveats**
Fried et al., 1997	OPPS	*General intelligence* [WISC-III, full scale IQ, verbal IQ, performance IQ, verbal comprehension index and (information, similarities and vocabulary) subtests]; *Academic achievement* (WRAT-R, single word reading recognition subtest; Woodcock reading mastery test, passage comprehension subtests); *Auditory comprehension/processing* {PPVT-R form L; Oral cloze test; [Regular and exception pseudoword task, (Composite score composed of phonological and orthographic subtests)]}; *Oral fluency* (Fluency test); *Auditory attention/concentration/discrimination* (Seashore rhythm test) **Total no. of outcome measures** **=** 48	9–12 year old children of women who reported MJ use during pregnancy (heavy MJ: *N* = 20; moderate MJ: *N* = 11; infrequent-no use: *N* = 100)	MJ use self-reported throughout pregnancy **Categories:** Infrequent-no use = AWJ < 1 Moderate = 1 ≤ AWJ < 6 Heavy = AWJ ≥ 6	No differences were observed	Relatively small number of participants studied in the MJ-exposed group Participants in MJ exposure group were not compared to an appropriate control group (AWJ = 0) Mothers reported tobacco cigarette smoking, alcohol, and cocaine use, which makes it difficult to assess the effect of MJ Maternal MJ use was determined exclusively from self-report
Fried et al., 1998	OPPS	*General intelligence* [WISC-III, full scale IQ, verbal IQ, performance IQ, perceptual organization index, verbal comprehension index, freedom from distractibility index, processing speed index, and (information, similarities, arithmetic, vocabulary, comprehension, digit span, picture completion, coding, picture arrangement, block design, object assembly, symbol search and mazes) subtests]; *Oral fluency* (Fluency test); *Working memory* (Auditory working memory); *Manual dexterity/spatial memory/tactile discrimination* (Tactual performance task, total time); *Cognitive flexibility* (Category test, total errors); *Visuomotor skills* (DTVMI); *Impulsivity/sustained attention* [(Gordon delay task, total rewards, total responses and total efficiency ratio) and (Gordon vigilance task, correct and commissions)] **Total no. of outcome measures** = 60	9–12 year old children of women who reported MJ use during pregnancy (heavy MJ: *N* = 20; infrequent-moderate MJ: *N* = 19: CTL: *N* = 92)	MJ use self-reported throughout pregnancy **Categories:** Infrequent-moderate = 0 < AWJ < 6 Heavy = AWJ ≥ 6	Heavy MJ exposure associated with poorer performance on object assembly subtests^*^ (WISC-III) Heavy MJ exposure associated with better sustained attention (Gordon vigilance task; correct and commissions)^†^ and comprehension subtest (WISC-III)^†^ performance No other differences were observed	Adjusted cognitive scores were not compared against a normative data set. Thus, the clinical importance of findings could not be determined Mothers reported tobacco cigarette smoking, alcohol, and cocaine use, which makes it difficult to assess the effect of MJ Mothers who reported heavy MJ use (six or more joints per day) had lower levels of education than other groups. Average parental education was also lower in this group, which makes it difficult to assess the effect of MJ Relatively small number of participants studied in the MJ-exposed group Maternal MJ use was determined exclusively from self-report
Fried and Watkinson, 2000	OPPS	*Basic visuoperceptual functioning* (TVPS, perceptual quotient composed of visual discrimination, memory, spatial relations, form constancy, sequential memory, figure-ground, and closing subtests); *Visual attention/task shifting* (Trail making test, time A and time B); *Visuoperceptual* *organization/problem solving* [WISC-III, (Perceptual organization index composed of block design, object assembly, picture completion, and picture arrangement subtests), symbol search, mazes and coding subtests]; *Visuomotor coordination* (DTVMI); *Visual attention span/memory/sequencing* (Knox cube); *Memory* (WISC, digit span subtest), *and attention* (WISC, freedom of distractibility index) **Total no. of outcome measures** **=** 44	9–12 year old children of women who reported MJ use during pregnancy (heavy MJ: *N* = 21; infrequent-moderate MJ: *N* = 23; CTL: *N* = 102)	MJ use self-reported throughout pregnancy **Categories:** Infrequent-moderate = 0 < AWJ < 6 Heavy = AWJ ≥ 6	Prenatal MJ exposure associated with poorer performance on the object assembly subtest^*^ and the perceptual organization index^*^ (WISC-III) No other differences observed	Despite poorer performance on two measures, MJ-exposed participants performed in the normal range on all cognitive tests Mothers reported tobacco cigarette smoking, alcohol, and cocaine use, which makes it difficult to assess the effect of MJ Mothers who reported heavy MJ use (six or more joints per day) had lower levels of education than other groups. Average parental education was also lower in this group, but this was controlled for Relatively small number of participants studied in the MJ-exposed group Maternal MJ use was determined exclusively from self-report
Richardson et al., 2002	MHPCD	*Learning and memory* (WRAML, composite screening index composed of picture, design, and story memory, and verbal learning subtests); *Visual attention/task shifting* (Trail making test adult version, time A and time B); *Selective attention/flexibility/processing speed* (Stroop color/word interference test golden version, word t-score, color t-score and color/word t-score); *Visuomotor coordination* (Grooved pegboard test, time to insert with dominant hand and time to insert with non-dominant hand); *Attention/vigilance* (CPT-II, mean omission errors, mean commission errors, trial 3 omission errors, and trial 3 commission errors); *Attention/impulsivity/processing/motor control* (PACE, attention, impulsivity, information processing efficiency, and motor control) **Total no. of outcome measures** **=** 60	10 year old children of women who reported MJ use during: 1st trimester (heavy MJ: *N* = 85; light-moderate MJ: *N* = 163; CTL: *N* = 345), 3rd trimester (heavy MJ: *N* = 30; light-moderate MJ: *N* = 83; CTL: *N* = 480)	MJ use self-reported at 4th & 7th pregnancy months and at 24–28 h post-delivery **Categories:** Exposed by trimester Not heavy = ADJ < 0.89 Heavy = ADJ ≥ 0.89	**1st trimester:** Heavy MJ exposure associated with poorer performance on two subtests of the WRAML (composite index^*^ and design memory^*^) **2nd trimester:** MJ exposure associated with poorer performance on the CPT-II (more commission errors)^*^ No other differences observed	Cognitive scores were not reported or compared against a normative data set. Thus, the clinical importance of findings could not be determined Unclear if participants in the heavy MJ exposure group were compared to an appropriate control group (ADJ = 0) Mothers who reported MJ use had higher rate of heavy alcohol, tobacco, and cocaine use than CTL mothers, which makes it difficult to assess the effect of MJ Relatively small number of participants studied in the heavy MJ-exposure group Maternal MJ use was determined exclusively from self-report
Goldschmidt et al., 2004	MHCPD	*Academic achievement* (WRAT-R, reading, spelling, and arithmetic subtests) and (PIAT-R, reading comprehension subtest) **Total no. of outcome measures** **=** 15	10 year old children of women who reported MJ use during: 1st trimester (MJ: *N* = 253; CTL: *N* = 353), 2nd trimester (MJ: *N* = 127; CTL: *N* = 421), 3rd trimester (MJ: *N* = 116; CTL *N* = 490)	MJ use self-reported at 4th & 7th pregnancy months and at 24–28 h post-delivery **Categories:** Exposed by trimester Not heavy=ADJ < 1 Heavy = ADJ ≥ 1	**2nd trimester:** MJ exposure associated poorer academic achievement (PIAT-R: reading comprehension)^*^ No other differences were observed	Cognitive scores were not compared against a normative data set. Thus, the clinical importance of findings could not be determined Mothers reported tobacco cigarette smoking, and alcohol use, which makes it difficult to assess the effect of MJ Maternal MJ use was determined exclusively from self-report
Hurt et al., 2005	CHP	*Impulsivity/sustained attention* (Gordon distractibility task, total correct and total commissions) **Total no. of outcome measures** **=** 2	10 year old children of women who reported MJ use during pregnancy (MJ: *N* = unknown; CTL *N* = unknown)	MJ use self-reported at post-delivery period Also, maternal and infant urine was collected **Categories:** Exposed	No differences were observed	Cognitive data not reported Only one cognitive measure used to assess a specific domain Mothers reported other drug use. It is not clear whether this was controlled for Unknown number of participants in each group It is unclear whether maternal and infant urine were analyzed for cannabinoids
Hurt et al., 2009	CHP	*Overall cognitive function composite score composed of inhibitory/impulse control* (Counting Stroop test and Go/No-go task), *working memory* (CANTAB letter 2-back test and Spatial working memory task), *set shifting/flexibility* (CANTAB intra/extra dimensional shift task), *impulsivity/sustained attention* (Gordon delay task), *auditory comprehension/processing* (PPVT-III*), grammatical contrast comprehension* (Test for the reception of grammar), *spatial cognition* (Eckstrom rotation and Benton line orientation), *visual object and space perception* (Warrington and James' visual object and space perception task shape detection and Mooney's test of visual closure face perception), *and memory* (incidental word and face learning) **Total no. of outcome measures** **=** 1	12 year old children of women who reported MJ use during pregnancy (MJ: *N* = 15; CTL *N* = 105)	MJ use self-reported at post-delivery period Also, maternal and infant urine was collected **Categories:** Exposed	No differences were observed	Data for a total of 14 cognitive tests were converted into one composite score. Scores for the separate cognitive tasks was not reported. Relatively small number of participants studied in the MJ group Mothers reported tobacco cigarette smoking, alcohol, and cocaine use, which makes it difficult to assess the effect of MJ It is unclear whether maternal and infant urine were analyzed for cannabinoids
Lewis et al., 2010	CWRUS	*Language development* (TOLD-I3, sentence combining, picture vocabulary, word ordering, generals, grammatical comprehension and malapropisms subtests) *and phonological processing* [CTOPP, phonological awareness composite score (elision and blending words subtests), phonological memory composite score (memory for digits and non-words subtests), and rapid naming composite scores (rapid naming of colors, objects, digits, and letters subtests)] **Total no. of outcome measures** **=** 15	10 year old children of women who reported MJ use during pregnancy (MJ: *N* = unknown; CTL: *N* = unknown) Also, maternal and infant urine analyzed for cannabinoids; infant meconium was analyzed for THC	MJ use self-reported at post-delivery period **Categories:** Exposed	No differences were observed	Cognitive data not reported Mothers reported tobacco cigarette smoking, alcohol, and cocaine use, and exposure to lead, which makes it difficult to assess the effect of MJ Unknown number of children in each group
Carmody et al., 2011	DRWJ	*Attention and inhibitory control* (Yale child study center attention task, overall accuracy score, inhibition errors, and attention errors) **Total no. of outcome measures** **=** 12	9 and 11 year old children of women who reported MJ use during pregnancy (MJ: *N* = unknown; CTL: *N* = unknown) Also, infant meconium was analyzed for THC	MJ use self-reported at either prenatally, at post-delivery period or in mother's home within 2 weeks of child's birth **Categories:** Exposed and exposed by gender	No differences were observed	Cognitive data not reported Mothers reported tobacco cigarette smoking, alcohol and cocaine use, which makes it difficult to assess the effect of MJ Unknown number of children in each group
Day et al., 2011	MHPCD	*General intelligence* (SBIS-IV, composite score) **Total no. of outcome measures** **=** 1	10 year old children of women who reported MJ use during pregnancy (MJ: *N* = unknown; CTL: *N* = unknown)	MJ use self-reported at 4th & 7th pregnancy months and post-delivery **Categories:** Not heavy = ADJ < 0.89 Heavy = ADJ ≥ 0.89	No differences were observed	Adjusted cognitive data not reported Participants in the heavy MJ exposure group were not compared to an appropriate control group (ADJ = 0) Unknown number of children in each group
Rose-Jacobs et al., 2011	BHUS	*Executive function* {D-KEFS, [Color-word interference (inhibition completion time, inhibition total errors, and inhibition/switching total errors), design fluency (switching total correct and design accuracy percent), trail making number-letter switching (completion time and error analysis), word context (total consecutively correct and consistently correct ratio) and tower (total achievement) subtests]} **Total no. of outcome measures** **=** 10	12−14 year old children of women who reported MJ use during pregnancy (heavy MJ: *N* = 15; light MJ: *N* = 18; CTL *N* = 104)	MJ use self-reported throughout pregnancy and shortly after delivery Also, maternal and infant urine analyzed for cannabinoids; infant meconium was analyzed for cannabinoids **Categories:** Exposed	Moderate, but not heavy, MJ exposure associated with poorer executive functioning (Design fluency: total correct switching)^*^ No other differences were observed	Average children's scores for most D-KEFS subtests was below the normal range Mothers reported tobacco cigarette smoking, and alcohol use, which makes it difficult to assess the effect of MJ
Rose-Jacobs et al., 2012	BHUS	*Academic achievement* (WIAT-II, word reading, reading comprehension, pseudoword decoding, numerical operations, math reasoning, spelling, written expression, oral expression, and listening comprehension subtests); *General intelligence* (WISC-III) **Total no. of outcome measures** **=** 10	11 year old children of women who reported MJ use during pregnancy (MJ: *N* = 25; CTL *N* = 94)	MJ use self-reported throughout pregnancy and shortly after delivery Also, maternal and infant urine analyzed for cannabinoids; infant meconium was analyzed for cannabinoids **Categories:** Exposed	Prenatal MJ exposure associated with better academic performance (WIAT-II: spelling)^†^ No other differences were observed	Adjusted cognitive data not reported Relatively small number of participants studied in the heavy MJ group Mothers reported tobacco cigarette smoking, alcohol, and cocaine use, which makes it difficult to assess the effect of MJ

*ADJ, Average daily joints; AWJ, Average weekly joints; BHUS, Boston and Harvard Universities Study; CHP, Children's Hospital of Philadelphia Study; CPT/CPT-II, Continuous performance test versions I and II; CTL, Control group; CTOPP, Comprehensive test of phonological processing; CWRUS, Case Western Reserve University Study; D-KEFS, Delis-Kaplan Executive Function System; DRWJ, Drexel and Robert Wood Johnson Universities Study; DTVMI, Developmental test of visual-motor integration; IQ, Intelligence quotient; MHPCD, Maternal Health Practices and the Child Development Study; MJ, Marijuana group; NCRR, National Center for Research Resources; NIAAA, National Institute on Alcohol Abuse and Alcoholism; NIDA, National Institute on Drug Abuse; OPPS, Ottawa Prenatal Prospective Study; PACE, Pediatric assessment of cognitive deficiency; PIAT-R, Peabody individual achievement test-revised; PPVT-III/PPVT-R, Peabody picture vocabulary test 3rd and revised editions; SBIS-IV, Stanford-Binet intelligence scale 4th edition; THC, Tetrahydrocannabinol; TOLD-I3, Test of language development-intermediate third edition; TVPS, Test of visual-perceptual skills; WIAT-II, Wechsler individual achievement test 2nd edition; WISC/WISC-III, Wechsler intelligence scale for children 1st and 3rd editions; WRAML, Wide range assessment of memory and learning; WRAT-R, Wide range achievement test-revised*.

* *Negative associations on cognitive outcomes*,

† *Positive associations on cognitive outcomes*.

Performance on the majority of 278 cognitive outcomes (96%) was similar between the groups. Prenatal cannabis exposure was significantly associated with 12 cognitive outcomes: better performance on four (Fried et al., 1998; Rose-Jacobs et al., 2012) and worse performance on eight (Fried et al., 1998; Fried and Watkinson, 2000; Richardson et al., 2002; Goldschmidt et al., 2004; Rose-Jacobs et al., 2011). The authors compared cognitive performance scores to a normative database in one of the five studies in which a significant association was found (Rose-Jacobs et al., 2011).

Richardson et al. (2002) assessed the cognitive functioning of 10-year-old children (maximum *N* = 163) whose mothers reported cannabis use during pregnancy. Of the 60 cognitive outcomes assessed, three were significantly associated with prenatal cannabis exposure. Exposure to more than 0.89 average daily joints in the 1st trimester was associated with poorer performance on the composite index and design memory subtests of the Wide Range Assessment of Memory and Learning, and overall exposure during the 2nd trimester was associated with more commission errors on the Continuous Performance Test—Version II (CPT-II). Again, cognitive scores were not reported or compared against a normative.

The findings above seem to conflict with those from a subsequent study. Rose-Jacobs et al. (2012) assessed the association between cognitive functioning and prenatal cannabis exposure in 11-year old children (maximum *N* = 18). Of the 10 cognitive outcomes assessed, one significant association was found. Prenatal cannabis exposure was associated with *better* spelling scores. Although cognitive task scores were reported they were not compared to a normative database.

In a 1998 investigation, Fried and colleagues assessed the executive functioning of 9- to 12-year old participants, who were exposed to cannabis prenatally (maximum *N* = 20) (Fried et al., 1998). Of the 60 cognitive outcomes assessed four significant associations were found. Children whose mothers smoked over 6 joints per week during pregnancy demonstrated *better* sustained attention both on the Gordon Vigilance task as evidenced by more correct responses and fewer commission errors and WISC-III comprehension subtest. However, the same level of exposure was associated with lower scores on the WISC-III's object assembly subscales. Although mean task scores were reported, they were not compared against a normative database. We attempted to obtain normative scores but were unable to do so, making it difficult to determine the clinical relevance of the findings.

In a subsequent study, Fried and Watkinson (2000) examined the cognitive functioning of 9- and 12-year-old children whose mothers reported cannabis use during pregnancy (maximum *N* = 23). Of the 44 cognitive outcomes assessed, two significant associations with prenatal cannabis exposure were found. Children prenatally exposed to more than 6 average weekly joints performed more poorly on the object assembly subtest and the perceptual organization index. Although mean task scores were reported, they were not compared against a normative database by the authors. We obtained normative scores and conducted the appropriate comparison. We found that mean scores for the object assembly subtest and perceptual organization index were within the normal range.

Goldschmidt et al. (2004) assessed the potential association between prenatal cannabis exposure and cognitive functioning by comparing over two hundred (maximum *N* = 253) 10-year-old children with prenatal cannabis exposure to matched controls. Of the 15 cognitive outcomes assessed, one significant association with prenatal cannabis exposure was found. Second trimester exposure was related to lower reading comprehension and underachievement. Although mean scores were reported, they were not compared to a normative database by the researchers. We attempted to obtain normative scores but were unable to do so, making it difficult to determine the clinical relevance of the findings. Regarding the underachievement (defined as a significant disparity between a child's score on the WRAT-R and the expected level of achievement based on SBIS scores), 15 percent (19/127) of the children exposed to more than one joint per day during the second trimester were classified as underachievers, compared with eight percent (33/421) among controls.

Unlike the above studies, Rose-Jacobs et al. (2011) compared cognitive task scores to a normative database. Of the 10 cognitive outcomes assessed, one significant association was found. After adjusting for covariates such as IQ and other drug use, lighter, but not heavier, prenatal cannabis exposure was associated with worse performance on the Design Fluency task (total correct switching). Importantly, Rose-Jacobs et al. (2011) determined that the mean score for this task fell within the normal range when compared against normative data.

#### Studies Assessing Adolescents and Early Adults (13 to 22 Years)

Table 4 shows studies assessing the cognitive functioning of adolescents and young adults. The MHPCD provided the majority (63 percent) of the cognitive outcomes. The OPPS contributed the rest (37 percent).

**Table 4:** Studies assessing cognition in adolescence and early adulthood (13 to 22 years).

**Investigators**	**Cohort**	**Domain tested**	**Participants**	**Exposure**	**Findings**	**Caveats**
Fried and Watkinson, 2001	OPPS	*Mirsky's five-factor model of attention {Attention/vigilance* (CPT); *Set-shifting/flexibility* (WCST-CV2); *Working memory* (WISC-III, arithmetic subtest); *Auditory memory* (Sentence memory test), *Auditory attention/concentration/discrimination* (Seashore rhythm test), *Visual attention span/memory/sequencing* (Knox cube); *Visuoperceptual organization/problem solving* [WISC-III, (Picture arrangement, arithmetic, block design and vocabulary subtests, transformed into Wechsler short form deviation quotient)]} **Total no. of outcome measures** **=** 22	13–16 year old children of women who reported MJ use during pregnancy (heavy MJ: *N* = 25; infrequent-moderate MJ: *N* = 26; CTL: *N* = 101)	MJ use self-reported throughout pregnancy **Categories:** Infrequent-moderate = 0 < AWJ < 6 Heavy = AWJ ≥ 6	Heavy prenatal MJ exposure was associated with poorer performance on one of five CPT measures (stability)^*^ No other differences were observed	Cognitive scores were not reported or compared against a normative data set. Thus, the clinical importance of findings could not be determined Mothers reported tobacco cigarette smoking, alcohol, and cocaine use, which makes it difficult to assess the effect of MJ Mothers who reported heavy MJ use (six or more joints per day) had lower levels of education than other groups. Average parental education was also lower in this group, but this was controlled for Children of mothers who reported MJ more likely to be exposed to postnatal 2nd hand cigarette smoke and be current cigarette smokers but this was controlled for. Unclear whether other substance use was assessed Small number of participants studied in the heavy MJ group Maternal MJ use was determined exclusively from self-report
Fried et al., 2003	OPPS	*Academic achievement* (WRAT, reading, spelling, and arithmetic subtests; PIAT, spelling recognition subtest); *Visual memory* (Abstract designs test, errors, and latency); *Visual attention span/memory/sequencing* (Knox cube); *Set-shifting/flexibility* (WCST-CV, perseverative errors), *Auditory memory* (Sentence memory test; Missing numbers test, errors); *Visuoperceptual organization/problem solving* (WISC-III short form, estimate of full-scale IQ); *Selective attention/flexibility/processing speed* (Stroop color and word test, interference score) **Total no. of outcome measures** **=** 12	13–16 year old children of women who reported MJ use during pregnancy (heavy MJ: *N* = 25; none-light MJ: *N* = 120)	MJ use self-reported throughout pregnancy **Categories:** None/light = AWJ < 6 Heavy = AWJ ≥ 6	Heavy prenatal MJ exposure was associated with lower spelling recognition scores (PIAT)^*^ and slower response times on a visual memory test (Abstract designs test) ^*^ No other differences were observed	PIAT: MJ-exposed participants scores remained within the normal range Participants in the heavy MJ exposure group were not compared to an appropriate control group (AWJ = 0) Mothers reported tobacco cigarette smoking, alcohol, and cocaine use, which makes it difficult to assess the effect of MJ Mothers who reported heavy MJ use (six or more joints per day) had lower levels of education than other groups. Average parental education was also lower in this group, but this was controlled for Children of mothers who reported MJ more likely to be exposed to postnatal 2nd hand cigarette smoke and some (25/145) were current cigarette smokers, but this was controlled for. Unclear whether other substance use was assessed Maternal MJ use was determined exclusively from self-report
Smith et al., 2004	OPPS	*Impulse control* (Go/No-go task; fMRI) The task yielded 6 outcome measures: (1) Errors of omission (Press for X and Press for all letters except X); (2) Errors of commission (Press for X and Press for all letters except X); and (3) Reaction Time, measured in seconds (Press for X and Press for all letters except X). **Total no. of outcome measures** **=** 6	18–22 year old children of women who reported MJ use during pregnancy (MJ: *N* = 16; CTL: *N* = 15)	MJ use self-reported throughout pregnancy **Categories:** Exposed = 0 < AWJ < ~8	Prenatal MJ exposure group committed more errors of commission for the “Press for all letters except X” condition^*^ No other performance differences were observed	Cognitive data not compared against normative data set. Thus, the clinical importance of findings could not be determined Some (13/31) participants tested positive for MJ or cocaine (2/31) and had smoked MJ on the morning of the testing day (4/31). It is unclear whether those reporting MJ or cocaine use were part of the MJ-exposed or non-exposed group, or how this was controlled for. Participants also reported alcohol and tobacco use, which makes it difficult to assess the effect of MJ Mothers reported tobacco cigarette smoking, alcohol, and caffeine use, which makes it difficult to assess the effect of MJ Small number of participants studied Sex differences in group composition: MJ-exposed group included 6 males and 10 females; Non-exposed group included 10 males and 5 females Maternal MJ use was determined exclusively from self-report
Smith et al., 2006	OPPS	*Visuospatial working memory* (Modified n-back task; fMRI); *General intelligence* (WISC, full scale IQ; WAIS, full scale IQ) *Visuospatial working memory* (Modified n-back task) The tasks yielded 6 outcome measures: (1) Errors of omission (Match to center and Press for 2-back); (2) Errors of commission (Match to center and Press for 2-back); and (3) Reaction Time, measured in seconds (Match to center and Press for 2-back). **Total no. of outcome measures** **=** 8	18–22 year old children of women who reported MJ use during pregnancy (MJ *N* = 16, CTL *N* = 15)	MJ use self-reported throughout pregnancy **Categories:** Exposed	No performance differences were observed	Some (13/31) participants tested positive for MJ. It is unclear whether those reporting MJ use were part of the MJ-exposed or non-exposed group. Participants also reported alcohol and tobacco use, which makes it difficult to assess the effect of MJ Mothers reported tobacco cigarette smoking, alcohol, and caffeine use, which makes it difficult to assess the effect of MJ Small number of participants studied Sex differences in group composition: MJ-exposed group included 6 males and 10 females; Non-exposed group included 10 males and 5 females Maternal MJ use was determined exclusively from self-report questionnaire
Willford et al., 2010	MHPCD	*Processing speed* (BCT, average unimanual speed, 0° angle completion time, 90° angle completion time, average bimanual symmetrical time, 45° symmetrical angle completion time, 135° symmetrical angle completion time, average bimanual asymmetrical time, 22.5° for left dominant or 67.5° for right dominant asymmetrical angle completion time, and 157.5° for left dominant or 112.5° for right dominant angle completion time), *visuomotor coordination* (BCT, average speed to complete 45/135° angles/unimanual speed, average speed to complete 22.5/157.5° angles/unimanual speed, average speed to complete 67.5 °/112.5° unimanual speed, 45° symmetrical angle reaction time, 135° symmetrical angle reaction time, 22.5° for left dominant or 67.5° for right dominant asymmetrical angle reaction time and 157.5° for left dominant or 112.5° for right dominant angle reaction time), *and interhemispheric transfer* (BCT, average left dominant reaction time, average right dominant reaction time, 22.5° for left dominant or 67.5° for right dominant asymmetrical angle reaction time and 157.5° for left dominant or 112.5° for right dominant asymmetrical angle reaction time) **Total no. of outcome measures** **=** 57	16 year old children of women who reported MJ use during: 1st trimester (MJ: *N* = 132; CTL: *N* = 188), 2nd trimester (MJ: *N* = 66; CTL: *N* = 226), 3rd trimester (MJ: *N* = 46; CTL *N* = 225)	MJ use self-reported at 4th & 7th pregnancy months and at birth **Categories:** Exposed by trimester	**1st trimester:** Prenatal MJ exposure associated with slower processing speed (22.5° for left dominant asymmetrical angle completion time)^*^ **3rd trimester:** Prenatal MJ exposure associated with better performance on measure of visuomotor coordination (135° symmetrical angle reaction time)^†^ and poorer performance on two measures of interhemispheric transfer (slower left dominant reaction time^*^ and 157.5° for left dominant asymmetrical angle reaction time^*^)	Cognitive data not reported or compared against normative data set. Thus, the clinical importance of findings could not be determined It is unclear whether those reporting MJ use were part of the MJ-exposed or non-exposed group Mothers reported tobacco cigarette smoking and alcohol use, which makes it difficult to assess the effect of MJ Maternal MJ use determined exclusively from self-report
Goldschmidt et al., 2012	MHPCD	*Academic achievement* (WIAT screener, composite score composed by basic reading, mathematics, and spelling) **Total no. of outcome measures** **=** 12	14 year old children of women who reported MJ use during: 1st trimester (heavy MJ: *N* = 79; light-moderate MJ: *N* = 139: CTL: *N* = 306); 2nd trimester (heavy MJ: *N* = 27; light-moderate MJ: *N* = 91: CTL: *N* = 361); 3rd trimester (heavy MJ: *N* = 34; light-moderate MJ: *N* = 74: CTL: *N* = 416)	MJ use self-reported at 4th & 7th pregnancy months and at 24–28 h post-delivery **Categories:** Exposed by trimester Not heavy = 0 ≤ ADJ < 1 Heavy = ADJ ≥ 1	**1st trimester:** Heavy prenatal MJ exposure associated with lower academic achievement scores (composite^*^ and basic reading^*^) No other differences were observed	Adjusted cognitive data not reported or compared against normative data set. Thus, the clinical importance of findings could not be determined Participants in the heavy MJ exposure group were not compared to an appropriate control group (ADJ = 0) Some (102) participants tested positive for MJ. It is unclear whether those reporting MJ use were part of the MJ-exposed or non-exposed group. Unclear whether other substance use was assessed Mothers reported tobacco cigarette smoking, and alcohol use, which makes it difficult to assess the effect of MJ Maternal MJ use determined exclusively from self-report
Richardson et al., 2015	MHPCD	*General intelligence* (WISC-III short form, estimated IQ); *Problem solving* (WISC-III, mazes subtest); *Abstract reasoning and executive function* (Children's category test level 2); *Memory* (Children's memory scale, verbal and visual memory, short- and long-term memory, recall, recognition, and working memory) **Total no. of outcome measures** = 30	15 year old children of women who reported MJ use during pregnancy (MJ *N* = unknown, CTL *N* = unknown)	MJ use self-reported at 7th pregnancy months and at 24–48 h post-delivery **Categories:** Exposed by trimester	No differences were observed	Cognitive scores were not reported Study population included children with WISC-III test scores below the normal range (IQ < 85; range 47–129). Thus, even if test scores for MJ and CTL groups would had been compared, the clinical importance of findings could not have been determined Mothers reported tobacco cigarette smoking, alcohol, cocaine and other drug use, which makes it difficult to assess the effect of MJ Unknown number of participants in MJ and CTL groups
Smith et al., 2016	OPPS	*General intelligence* (WAIS, full scale IQ); *Visuospatial working memory* (Modified n-back task, fMRI); *Impulse control* (Go/No-go task, fMRI) task behavioral data were previously reported (Smith et al., 2004, 2006); *Visuospatial working memory* (Letter 2-back task) The tasks yielded 6 outcome measures: (1) Errors of omission (Press for X and Press for all letters except X); (2) Errors of commission (Press for X and Press for all letters except X); and (3) Reaction Time, measured in seconds (Press for X and Press for all letters except X). *Inhibitory Control* (Counting Stroop test, fMRI) The tasks yielded 4 outcome measures: (1) Errors of commission (congruent and incongruent); and (3) Reaction Time, measured in seconds (congruent and incongruent). **Total no. of outcome measures** = 10	18–22 year old children of women who reported MJ use during pregnancy (MJ *N* = 16, CTL *N* = 15)	MJ use self-reported throughout pregnancy **Categories:** Heavy throughout entire pregnancy (AWJ ≥ 8)	No differences were observed	Some participants tested positive for MJ. It is unclear whether those reporting MJ use were part of the MJ-exposed or non-exposed group. Participants also reported alcohol and tobacco use but this was controlled for Mothers reported tobacco cigarette smoking, alcohol, and caffeine use, which makes it difficult to assess the effect of MJ Small number of participants studied Sex differences in group composition: MJ-exposed group included 6 males and 10 females; Non-exposed group included 10 males and 5 females Maternal MJ use was determined exclusively from self-report questionnaire

*ADJ, Average daily joints, AWJ, Average weekly joints, BCT, Bimanual coordination task; CPT, Continuous performance test version I; CTL, Control group; fMRI, Functional magnetic resonance imaging; IQ, Intelligence Quotient; MHPCD, Maternal Health Practices and the Child Development Study; MJ, Marijuana group; NIAAA, National Institute on Alcohol Abuse and Alcoholism; NIDA, National Institute on Drug Abuse; OPPS, Ottawa Prenatal Prospective Study; PIAT, Peabody individual achievement test; WAIS, Weschler adult intelligence scale; WCST-CV/WCST-CV2, Wisconsin card sorting test-computer versions I and II; WIAT, Wechsler individual achievement test 1st edition; WISC/WISC-III, Wechsler intelligence scale for children 1st and 3rd edition; WRAT, Wide range achievement test*.

* *Negative associations on cognitive outcomes*,

† *Positive associations on cognitive outcomes*.

Performance on the majority (94%) of the 157 cognitive outcomes assessed was similar between the groups. However, prenatal cannabis exposure was associated with better performance on one cognitive outcome (Willford et al., 2010) and worse performance on nine (Fried and Watkinson, 2001; Fried et al., 2003; Smith et al., 2004; Willford et al., 2010; Goldschmidt et al., 2012). For this age group, participant's cognitive scores were not compared to norms in any of the studies that found significant associations with prenatal cannabis exposure (Table A4 in Supplementary Material).

Willford et al. (2010) assessed the cognitive functioning of 16-year-old children whose mothers reported cannabis use during pregnancy (maximum *N* = 132). Of 57 cognitive outcomes assessed, four significant associations with prenatal cannabis exposure were found. Cannabis exposure in the 3rd trimester was associated with *better* performance on a measure of visuomotor coordination. In addition, cannabis exposure in the 1st trimester was associated with slower processing speed and with two measures of interhemispheric transfer in the 3rd trimester on the Bimanual Coordination Task (BCT). Children whose mothers smoked cannabis during the 3rd trimester of pregnancy also demonstrated *better* BCT performance on a measure of visuomotor coordination. It is important to point out that task scores were not reported and that normative data are not available for the tests used.

The finding of *better* visuomotor coordination above seems to conflict with findings from an earlier study. In that study, Smith et al. (2004) employed fMRI in young adults that were (*N* = 16) or were not (*N* = 15) prenatally exposed to cannabis while they completed a Go/No-Go task. Of the six cognitive outcomes assessed, one significant association with prenatal cannabis exposure was found. Participants exposed to cannabis prenatally had significantly more errors of commission for the “Press for all letters except X” condition than did controls. Of note, the clinical significance of this finding could not be determined because task scores were not reported and norms were not available.

Fried and Watkinson (2001) assessed the cognitive functioning of children of women who reported cannabis use during pregnancy (maximum *N* = 26). Of 22 cognitive outcomes assessed, one significant association with prenatal cannabis exposure was found. Prenatal exposure to more than 6 average weekly joints was associated with poorer performance on the CPT stability subtest. Task scores were not reported or compared against a normative dataset making it difficult to understand the clinical relevance of the finding.

Goldschmidt et al. (2012) did report mean cognitive task scores of children of women who reported cannabis use during pregnancy (maximum *N* = 139). Of the 12 cognitive outcomes assessed, two significant associations with prenatal cannabis exposure were found. Children exposed to one or more joints per day during the first trimester achieved lower Wechsler Individual Achievement Test (WIAT) composite and reading scores. Although unadjusted mean cognitive task scores were reported, they were not compared against normative data. We attempted to obtain normative scores but were unable to do so, making it difficult to determine the clinical relevance of the finding. It is also important to note that it is unclear whether other illicit drug use was assessed in the study.

Fried et al. (2003) assessed cognitive functioning of 13 to 16-year-old children exposed to cannabis prenatally (maximum *N* = 25). The researchers also controlled for mothers' other illicit drug use. Of the 12 cognitive outcomes assessed, two significant associations with prenatal cannabis exposure were found. Prenatal exposure to more than 6 weekly joints was associated with slower reaction times on the abstract designs test and lower spelling recognition scores on the Peabody individual achievement test (PIAT). Regarding the Abstract Designs Test, task scores were not reported or compared against a normative dataset. On the other hand, task scores were reported for the PIAT. We obtained norms for the task and found that they were within the normal range.

## Discussion

### General Discussion of Findings

In general, the findings of this critical review indicate that prenatal cannabis exposure is associated with few effects on the cognitive functioning of offspring. Overall, we found a total of 1,001 statistical comparisons between groups of participants that were exposed to cannabis prenatally and non-exposed controls. Cognitive performance was statistically different on only 4.3% of cognitive measures—worse on 3.4% and better in 0.9%. Importantly, we found evidence for scores being below the normal range in only 0.3% of the total sample. Thus, despite analyzing studies spanning approximately three decades, we conclude the evidence does not support an association between prenatal cannabis exposure and clinically relevant cognitive deficits.

Nonetheless, the study by Goldschmidt et al. (2008) deserves special attention as it is the only article we found where the group exposed to cannabis prenatally obtained scores that fell outside the normal range of cognitive functioning. The authors concluded that prenatal cannabis exposure “has a significant effect on school-age intellectual development” and “could impair a child's academic functioning.”

The above conclusion should probably be tempered for several reasons. First, there were differences in maternal cognitive ability, poverty, and home environment. This is critical, as it has been demonstrated that poverty adversely affects children's cognitive development (Hurt and Betancourt, 2016). Although controlled for statistically, it is difficult to account for the potential effects of these covariates. Second, contributions of preschool and day-care attendance were not determined because this information was not available for all participants. This makes it difficult to determine the extent to which the current findings overlap with those of an earlier investigation by this group of researchers, which found that preschool and day-care attendance mediated the relationship between prenatal cannabis exposure and cognitive functioning (Day et al., 1994). More importantly, it precludes deeper analysis into variables that may have greater explanatory power than cannabis exposure. Third, only unadjusted scores were reported, and therefore, we were only able to compare these to a normative database. It remains to be determined if after scores are adjusted for sociodemographic and other factors they might be within the normal range.

### Assessing Cognitive Function

Another limitation in the study by Goldschmidt et al. (2008), is that only one task was used to measure each domain. This limitation was observed in virtually all other studies. Performance on multiple tasks, which assess the same domain, should be evaluated in neuropsychological research because individual tasks may tap slightly different components of the domain of interest. This can also help clarify interpretations when conflicting results are obtained with only one measure.

The study by O'Connell and Fried (1991) is a good case in point. The researchers used multiple measures allowing for a more comprehensive understanding of the impact of cannabis exposure on the domains assessed. The authors compared 28 prenatally exposed children (6–9 years old) with 28 non-exposed children (O'Connell and Fried, 1991). Assessment was comprehensive, tapping multiple domains including attention, cognitive flexibility and memory for a total of 27 cognitive outcomes. Participants were matched on the basis of maternal prenatal alcohol and tobacco consumption. Mothers of children exposed prenatally had consumed on average more than 1 joint per week, but exposure ranged from one to 50 joints per week (mean = 14, SD = 15). Prenatally exposed children performed as well as controls, and no positive or negative associations were found on cognitive measures. There are now multiple studies assessing children of similar ages that agree with these results (Hayes et al., 1991; Fried et al., 1992b; Noland et al., 2003b, 2005; Frank et al., 2005; Beeghly et al., 2006; Morrow et al., 2006; Mayes et al., 2007; Bennett et al., 2008; Carmody et al., 2011). In the future, researchers should keep in mind that meaningful group differences should be observed on multiple measures of a particular cognitive domain before making assertions about the long-term impact of prenatal cannabis exposure.

### Determining Clinical Significance and Language Precision

When we attempted to determine the extent to which cognitive performance was truly impaired—fell below the average range when compared against a normative database—we found evidence for this in only 3 of the 1,004 possible cognitive outcomes measured (<0.3% percent).

Despite this, there appears to be a tendency to interpret any difference as deficits representing substantial loss of function. One possible reason for confusion might be that the term “deficit” has at least two meanings and some conflate them. One is captured by the canonical situation in which one group performs statistically significantly less well on a task. However, its clinical significance, or everyday import, is difficult or impossible to determine because scores are rarely compared against a normative database. Normative data are obtained from a large, randomly selected representative sample and incorporate important variables such as age and education, and establish a baseline distribution for a measurement. Scores obtained empirically should be compared against norms to determine whether statistically significant findings are meaningful. This brings us to the second meaning of the term “deficit,” a substantial loss of function, in which performance falls outside of the normal range and bears clinical significance (Both meanings probably represent end points on a continuum). The problem in the literature on prenatal substance exposure is that in most studies, results support only the first (or difference) interpretation, but are discussed in terms of the “dysfunctional” interpretation. In essence, the English word “deficit” (or “impairment”) is ambiguous, and researchers in this field often switch meanings in moving from actual findings to discussion of the implications of such findings.

The majority of the reviewed studies did not include a comparison with normative data. In many cases, mean task scores were not reported making it impossible for others to make comparisons. Researchers should be encouraged to report data obtained for each individual participant, and use measures for which normative data has been collected and is available. This will go a long way in determining the extent to which prenatal cannabis exposure affects subsequent cognition or any other measure. Unfortunately, simply stating that there is a deficit in one group does not adequately inform future research, nor does it provide useful guidance for public policy makers, legal practitioners, or health professionals.

Previously, other researchers in this area have also cautioned against making definitive statements about “deficits” (Fried and Smith, 2001; Fried, 2002; Huizink and Mulder, 2006). Fried and Smith (2001) felt so strongly about this that they remarked, “caveats, coupled with the relatively sparse literature, are a combination that makes any definitive statement problematic, presumptuous, and foolhardy” (Fried and Smith, 2001). Unfortunately, the literature is replete with language purporting to report the “impact of prenatal cannabis exposure,” when what is actually reported is merely a correlational relationship (a positive or negative association). The inappropriate use of causal language tends to lead to premature and/or erroneous conclusions regarding the actual effects of prenatal cannabis exposure on subsequent cognitive functioning.

### Examination and Reporting of Confounding Variables

A greater understanding is necessary of the fact that many children with prenatal cannabis exposure are also exposed to factors often seen in people with low socio-economic status, such as poor nutrition, parents with lower levels of education and parents who may also use other substances, including nicotine and alcohol, among a host of other confounding variables. While a select few of the studies included in this review assessed and controlled for some of these variables, the majority did not. One noteworthy example is that tobacco use frequently occurred with cannabis use, and results from several studies that have revealed prenatal tobacco exposure alone, both directly through smoking by the mother and indirectly by secondhand smoke, is associated with lower scores in several cognitive domains (Polanska et al., 2015). These observations make it particularly difficult to disentangle the effects of tobacco from those of cannabis. Nonetheless, a consequence of the current movement to liberalize cannabis laws is that there appears to be a growing number of women who use cannabis-based products—and not other substances such as tobacco and alcohol—to treat nausea and vomiting during pregnancy (Dickson et al., 2018; Young-Wolff et al., 2019). This development provides an opportunity for future studies to assess the impact of prenatal cannabis exposure alone. Additional studies should account for the many other variables that potentially influence subsequent functioning of prenatally exposed offspring.

### Exposure: Timing, Amount, and Dose-Dependence

Only a limited number of studies confirmed the presence of cannabis with biological assays and none conducted quantitative analyses to confirm mother's self-reported cannabis use. In addition, few tested for the potential effects of prenatal cannabis exposure as a function of trimester. Both factors may be important for determining the extent to which prenatal cannabis exposure impacts cognitive functioning.

Another concern is the inappropriate use of the term “dose-response fashion.” This term was used by researchers to characterize that greater frequency of cannabis use was associated with a higher risk of adverse effects (Fried et al., 1992a; Fried and Watkinson, 2001). In a true dose-response study, however, all factors are held constant and experimenters systematically alter drug exposure. Increased effects with increasing dose are then a powerful demonstration that it is the drug that is causing effects. However, mothers who used more cannabis were different people—likely leading different lives—from those using less or no cannabis. Researchers made statistical corrections for as many covariates as they could, based on the measures included in the original studies, but it seems unlikely that these captured all of the complex differences expected among mothers and their children. In short, it is too strong a claim to describe results as a “dose-response” because: (1) all other factors were not held constant, and (2) the varied exposure to cannabis was not manipulated as a controlled experiment, but occurred for other, unknown, reasons.

### Type I and Type II Errors

Almost all studies in this literature have set their Type I error rate to 0.05, where stated. Given the number of negative and positive associations of prenatal cannabis exposure with cognitive outcomes compared to comparison groups, there is a strong likelihood of both Type I and Type II errors. The findings of this review, namely that only 3 of the 1,004 possible cognitive outcomes measured (<0.3% percent) fell below the average range when compared against a normative database, are what we would statistically expect to find only by chance.

### Limitations

The current review has at least three important limitations. First, as described, we acknowledge that normative data were unavailable for a limited number of measures on which prenatally exposed children performed significantly worse than controls. Therefore, it is possible that some of these measures were, in fact, below the normal range and might have changed our conclusions. In order to address this issue, future studies should either conduct the appropriate comparisons or report individual participants' scores and the normal range for each cognitive task so that readers can draw appropriate conclusions.

Another potential drawback is that its focus is limited to cognitive functioning. This focus was selected because of the obvious clinical implications and because it is within the realm of the authors' expertise area. We recognize, however, that there is a wide range of possible effects of prenatal cannabis exposure on the developing fetus, including effects on birth weight and emotional development. As a result, it is our hope that researchers critically review the influence of early cannabis exposure on other outcome measures.

In addition, it is possible that the cognitive tasks employed by most of the investigators of the reviewed studies are insensitive to the prenatal effects of cannabis. As such, more sensitive task batteries might result in a different pattern of findings. This possibility, along with other limitations associated with the current review, suggest that caution should be exercised when extrapolating findings from this investigation.

### Implications

The current review of the literature found that there are relatively few cognitive alterations noted in offspring exposed to cannabis prenatally. It is important also to note that these results should be interpreted taking into account limitations of the current state of the literature. It is also critical to understand that subsequent studies, especially those that address the limitations point out here, may yield a more concerning pattern of effects.

Regardless, at present, we are concerned that a misunderstanding of the relationship between prenatal cannabis exposure and subsequent cognitive functioning leads to an oversimplification of the complex relationships between socioeconomic factors and functioning of the individual whether drug use is involved or not. Misinterpretations of the complex interactions of relevant factors in itself can cause harm to pregnant women and their children by leading to punitive policies and enhancing unwarranted stigma. In some cases, intense stigma has resulted in removal of children from their families, and even in maternal incarceration. The rationale for such policies is, in part, that prenatal cannabis exposure causes persistent deleterious effects, especially on cognitive functioning. Findings from this review suggest that this assumption should be reevaluated to ensure that our assumptions do not do more harm than the drug itself.

## Author Contributions

CT: conceptualization, literature survey, statistical analyses, manuscript preparation and editing. CM-K and KO'M: data extraction, preparation of the tables, manuscript editing, and supporting role in conceptualization. CH: idea and conceptualization, literature survey, preparation of the first manuscript draft, manuscript editing, project leader, and corresponding author.

## Conflict of Interest

The authors declare that the research was conducted in the absence of any commercial or financial relationships that could be construed as a potential conflict of interest.

## Abbreviations

ADJ: Average daily joints
AWJ: Average weekly joints
BCT: Bimanual coordination task
BHUS: Boston and Harvard Universities Study
BSID: Bayley scales of infant development 1st edition
BSID-II: Bayley scales of infant development 2nd edition
CELF-3: Child evaluation of language fundamentals-third edition
CELF-P: Clinical evaluation of language fundamentals-preschool 1st edition
CHP: Children's Hospital of Philadelphia Study
CPT: Continuous performance test version I
CPT-II: Continuous performance test version II
CPT-III: Continuous performance test version III
CTL: Control group
CTOPP: Comprehensive test of phonological processing
CWRUS: Case Western Reserve University Study
D-KEFS: Delis-Kaplan Executive Function System
DRWJ: Drexel and Robert Wood Johnson Universities Study
DTVMI: Developmental test of visual-motor integration
fMRI: Functional magnetic resonance imaging
IQ: Intelligence quotient
MHPCD: Maternal Health Practices and the Child Development Study
MJ: Marijuana group
NCRR: National Center for Research Resources
NIAAA: National Institute on Alcohol Abuse and Alcoholism
NIDA: National Institute on Drug Abuse
OPPS: Ottawa Prenatal Prospective Study
PACE: Pediatric assessment of cognitive deficiency
PDT: Picture deletion task for preschoolers-modified
PIAT: Peabody individual achievement test
PIAT-R: Peabody individual achievement test-revised
PPVT-III: Peabody picture vocabulary test 3rd edition
PPVT-R: Peabody picture vocabulary test revised edition
SD: Standard deviation
SBIS-IV: Stanford-Binet intelligence scale 4th edition
THC: Tetrahydrocannabinol
TOLD-I3: Test of language development-intermediate third edition
TOLD-P3: Test of language development-primary third edition
TVPS: Test of visual-perceptual skills
UMJS: University of Miami's Jamaican Study
UMSM: University of Miami School of Medicine Study
U.S.: United States
WAIS: Weschler adult intelligence scale
WCST-CV: Wisconsin card sorting test-computer version I
WCST-CV2: Wisconsin card sorting test-computer version II
WIAT: Wechsler individual achievement test 1st edition
WIAT-II: Wechsler individual achievement test 2nd edition
WISC: Wechsler intelligence scale for children 1st
WISC-III: Wechsler intelligence scale for children 3rd edition
WISC-IV: Wechsler intelligence scale for children 4th edition
WISC-R: Wechsler intelligence scale for children revised edition
WPPSI-R: Wechsler preschool and primary scales of intelligence-revised
WRAML: Wide range assessment of memory and learning
WRAT: Wide range achievement test
WRAT-R: Wide range achievement test-revised
YCSC: Yale Child Study Center.
