# Supplementary material for: Totality of the Evidence Suggests Prenatal Cannabis Exposure Does Not Lead to Cognitive Impairments: A Systematic and Critical Review
Source: Front Psychol. 2020 May 8;11:816. doi: 10.3389/fpsyg.2020.00816 (PMC7225289; doi:10.3389/fpsyg.2020.00816)
Supplement: Supplementary file 1 [file Data_Sheet_1.pdf]

Table 1a. Clinical relevance in studies assessing cognition in infants and toddlers (up to 24 months)

**Investigators**	**Individual scores reported**	**Individual scores compared to normative data**	**Mean scores reported**	**Mean scores compared to normative data**	**Adjusted mean scores reported**	**Adjusted mean scores compared to normative data**
Fried and Watkinson, 1988✝	No	No	**Yes**	No<br>No negative associations reported	No	No
Richardson et al., 1995**	No	No	No	No	**Yes**<br>9 month olds/3rd trimester: BSID Mental development index<br>CTL= 123<br>Light= 124<br>Moderate=125<br>Heavy=112.4	**Yes**<br>Compared in original article
Singer et al., 1999	No	No	No	No	No	No
Singer et al., 2002	No	No	No	No	No	No
Noland et al., 2003a	No	No	No	No	No	No
Singer et al., 2005✝	No	No	No	No	No	No
Richardson et al., 2008	No	No	No	No	No	No

*Negative associations found on one or more cognitive outcomes, ✝Positive associations found on one or more cognitive outcomes

Table 2a. Clinical relevance in studies assessing cognition in children (3 to 9 years)

**Investigators**	**Individual scores reported**	**Individual scores compared to normative data**	**Mean scores reported**	**Mean scores compared to normative data**	**Adjusted mean scores reported**	**Adjusted mean scores compared to normative data**
Fried and Watkinson, 1990***✝	No	No	**Yes** Four year olds: McCarthy's Scales verbal CTL=64.2 Heavy=59.5 memory CTL=60.2 Heavy=54.2 PPVT-R vocabulary CTL=116.6 Heavy=109.3	No Normative data unavailable	**Yes/No** Four year olds: McCarthy's Scales memory CTL=60.1 Heavy=54.5	No Normative data unavailable
Hayes et al., 1991	No	No	No	No	No	No
O'Connell and Fried, 1991	No	No	**Yes**	No No negative associations reported	No	No
Fried et al., 1992a*	No	No	**Yes** Gordon vigilance task total correct CTL=31.1 Moderate=28.5 Heavy=25.5 omissions CTL=13.9	No Mean performance scores did not differ as a function of group	No	No

			Moderate=15.8<br>Heavy=19.4			
Fried et al., 1992b	No	No	**Yes**	No<br>No negative associations reported	No	No
Day et al., 1994**	No	No	No	No	No	No
Leech et al., 1999*✝	No	No	No	No	No	No
Noland et al., 2003b	No	No	**Yes/No**	No<br>No negative associations reported	No	No
Lewis et al., 2004*	No	No	No	No	No	No
Frank et al., 2005	No	No	No	No	No	No
Noland et al., 2005	No	No	No	No	No	No
Beeghly et al., 2006	No	No	No	No	No	No
Morrow et al., 2006	No	No	No	No	No	No
Mayes et al., 2007	No	No	No	No	No	No
Bennett et al., 2008	No	No	No	No	No	No
Goldschmidt et al., 2008*****	No	No	**Yes**<br>1st trimester: SBIS-IV verbal reasoning<br>CTL=101<br>Heavy=96<br>2nd trimester: SBIS-IV short-term memory<br>CTL=93<br>Heavy=86<br>quantitative reasoning	No<br>Compared by authors	No	No

			CTL=94<br>Heavy=84<br>composite score<br>CTL=92<br>Heavy=84<br>3rd trimester: SBIS-IV<br>quantitative reasoning<br>CTL=92<br>Heavy=86			
Singer et al., 2008*	No	No	No	No	No	No
Richardson et al., 2009**	No	No	No	No	No	No
Carmody et al., 2011	No	No	No	No	No	No

*Negative associations found on one or more cognitive outcomes, †Positive associations found on one or more cognitive outcomes

Table 3a. Clinical relevance in studies assessing cognition in early adolescence (9 to 12 years)

Investigators	Individual scores reported	Individual scores compared to normative data	Mean scores reported	Mean scores compared to normative data	Adjusted mean scores reported	Adjusted mean scores compared to normative data
Fried et al., 1997	No	No	**Yes**	No<br>No negative associations reported	**Yes**	No<br>No negative associations reported
Fried et al., 1998*†††	No	No	**Yes**<br>WISC-III<br>object assembly<br>CTL=11.4<br>Heavy=10.6	No<br>Norms not publicly available	**Yes**<br>WISC-III<br>object assembly<br>CTL=11.2<br>Heavy=10.4	No<br>Norms not publicly available
Fried and Watkinson, 2000**	No	No	**Yes**<br>WISC-III<br>object assembly<br>CTL=11.5<br>MJ=10.6<br>perceptual organization index<br>CTL=111.8<br>MJ=106.7	No<br>Compared by authors	**Yes**<br>WISC-III<br>object assembly<br>CTL=11.5<br>MJ=10.6<br>perceptual organization index<br>CTL=111.9<br>MJ=106.9	No<br>Compared by authors
Richardson et al., 2002***	No	No	No	No	No	No
Goldschmidt et al., 2004*	No	No	**Yes**<br>2nd trimester:<br>PIAT-R<br>Reading	No<br>Norms not publicly available	No	No

			comprehension<br>CTL=95.6<br>MJ=86 to 91.5			
Hurt et al., 2005	No	No	No	No	No	No
Hurt et al., 2009	No	No	No	No	No	No
Lewis et al., 2010	No	No	No	No	No	No
Carmody et al., 2011	No	No	No	No	No	No
Day et al., 2011	No	No	**Yes**	No<br>No negative associations reported	No	No
Rose-Jacobs et al., 2011*	No	No	**Yes**<br>Design fluency total correct switching<br>CTL=9.34<br>Moderate=8.28<br>Heavy=9.33	**Yes**<br>Compared in original article	**Yes**<br>Design fluency total correct switching<br>CTL=9.42<br>Moderate=7.89<br>Heavy=9.24	**Yes**<br>Compared in original article
Rose-Jacobs et al., 2012✝	No	No	**Yes**	No<br>No negative associations reported	No	No

*Negative associations found on one or more cognitive outcomes, ✝Positive associations found on one or more cognitive outcomes

Table 4a. Clinical relevance in studies assessing cognition in adolescence and early adulthood (13 to 22 years)

**Investigators**	**Individual scores reported**	**Individual scores compared to normative data**	**Mean scores reported**	**Mean scores compared to normative data**	**Adjusted mean scores reported**	**Adjusted mean scores compared to normative data**
Fried and Watkinson, 2001*	No	No	No	No	No	No
Fried et al., 2003**	No	No	No	No	No	No
Smith et al., 2004*	No	No	No	No	No	No
Smith et al., 2006	No	No	**Yes**	No<br>No negative associations reported	No	No
Willford et al., 2010***✝	No	No	No	No	No	No
Goldschmidt et al., 2012**	No	No	**Yes**<br>WIAT screener composite CTL=89.9 Heavy=83.9 basic reading CTL=93.8 Heavy=87.8	No<br>Norms not publicly available	No	No
Richardson et al., 2015	No	No	No	No	No	No
Smith et al., 2016	No	No	**Yes**	No<br>No negative associations reported	**Yes**	No<br>No negative associations reported

*Negative associations found on one or more cognitive outcomes, ✝Positive associations found on one or more cognitive outcomes
